# Adrenaline is a prominent driver of inflammatory responses following hypoglycaemia

**DOI:** 10.1007/s00125-026-06667-9

**Published:** 2026-01-29

**Authors:** Ilyas F. Mustafajev, Marijn S. Hendriksz, Rinke Stienstra, Cees J. Tack, Bastiaan E. de Galan, Rick I. Meijer

**Affiliations:** 1 Department of Internal Medicine, Radboud University Medical Center, Nijmegen, the Netherlands; 2 Division of Human Nutrition and Health, Wageningen University, Wageningen, the Netherlands; 3 Department of Internal Medicine, Maastricht University Medical Center (MUMC+), Maastricht, the Netherlands; 4 Cardiovascular Research Institute Maastricht (CARIM), Maastricht University, Maastricht, the Netherlands

**Keywords:** Adrenaline, Hypoglycaemia, Inflammation, Stress, Type 1 diabetes

## Abstract

**Aims/hypothesis:**

Hypoglycaemia induces an acute and sustained inflammatory response both in people with type 1 diabetes and in people without diabetes. To investigate the role of adrenaline (epinephrine) in this response, we measured the inflammatory effects of adrenaline in a concentration and timeframe comparable to that seen during hypoglycaemia, in people with type 1 diabetes and matched control participants without diabetes.

**Methods:**

Adults with type 1 diabetes and matched control participants received adrenaline intravenously at 0.04 µg kg^−1^ min^−1^ for 1 h. Blood was drawn at baseline, after 30, 60 and 180 min, and on days 1, 3 and 7 following start of adrenaline administration, to determine white blood cell counts, cytokine secretion using ex vivo stimulation of isolated monocytes and circulating inflammatory markers using the Olink inflammatory panel.

**Results:**

Adrenaline acutely increased neutrophil, lymphocyte and monocyte counts in both groups. While neutrophil and monocyte levels returned to baseline after 1 day, lymphocytes remained elevated for 7 days. Adrenaline acutely altered monocyte function towards a more inflammatory phenotype, reflected by increased secretion of cytokines after ex vivo stimulation with lipopolysaccharide in both groups. Adrenaline also increased circulating inflammatory proteins, including urokinase-type plasminogen activator, Fms-like tyrosine kinase 3 ligand, chemokine (C-X3-C motif) ligand 1 and fibroblast growth factor 21, after 7 days in both groups, with a more pronounced response in people with type 1 diabetes.

**Conclusions/interpretation:**

Levels of adrenaline similar to those seen in response to hypoglycaemia elicit an acute and prolonged inflammatory response in people with type 1 diabetes and matched control participants on a cellular, functional and protein level. These findings suggest that adrenaline is a prominent driver of inflammatory responses following hypoglycaemia.

**Trial registration:**

ClinicalTrials.gov NCT05990933

**Graphical Abstract:**

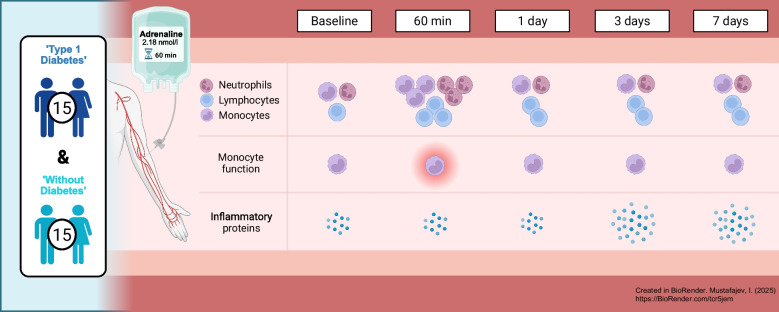

**Supplementary Information:**

The online version contains peer-reviewed but unedited supplementary material available at 10.1007/s00125-026-06667-9.


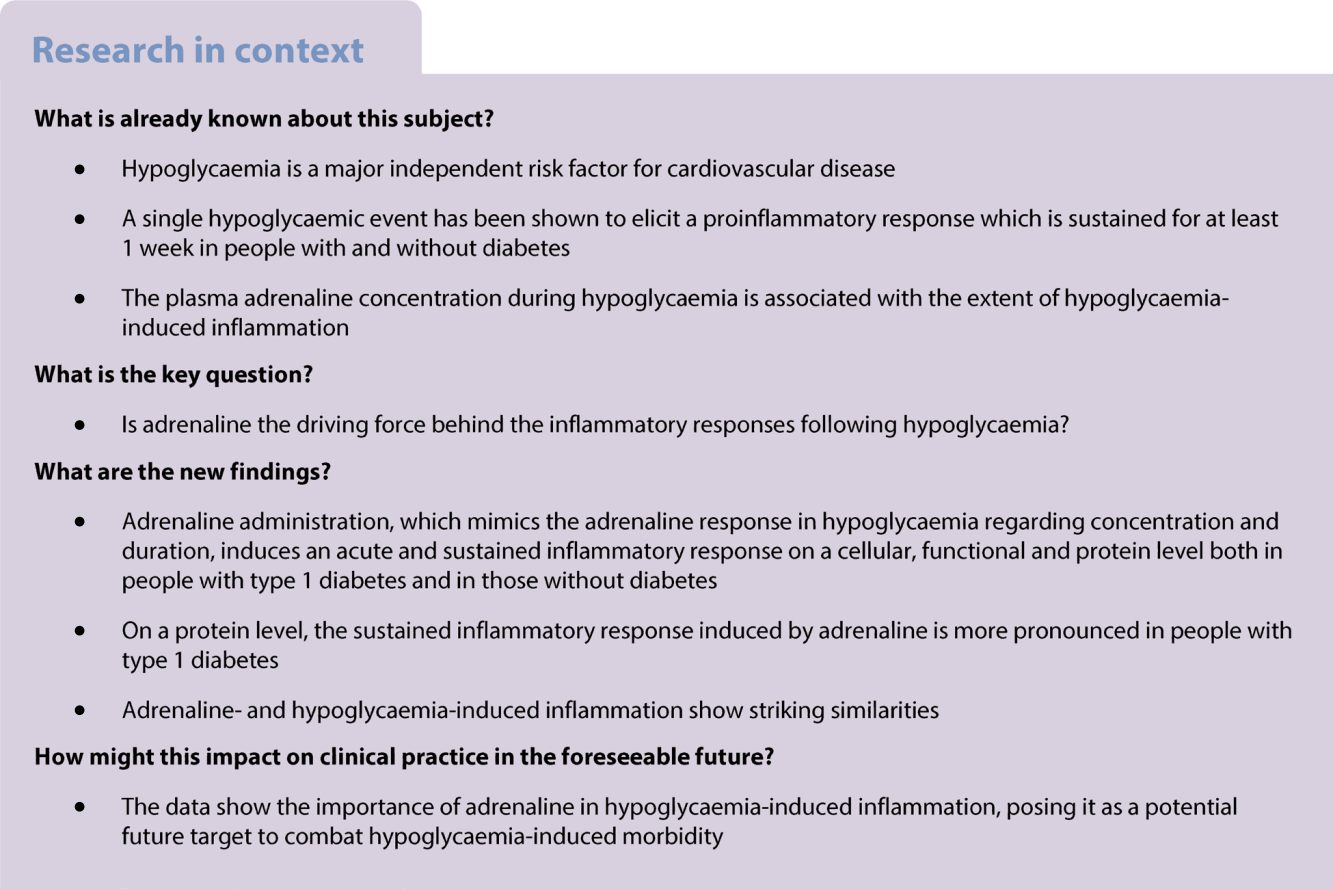


## Introduction

Type 1 diabetes is characterised by absolute insulin deficiency due to beta cell destruction in the pancreas. This insulin deficiency requires daily insulin administration to regulate glucose levels and prevent micro- and macrovascular complications [1]. The most common side effect of insulin therapy is hypoglycaemia, occurring 2–3 times per week on average, with severe events occurring once per year [2, 3]. Frequent hypoglycaemic episodes have been associated with a twofold increase in cardiovascular events, independent of other risk factors and despite achieving good glycaemic management [4, 5]. The main underlying cause of cardiovascular disease is atherosclerosis, with inflammation being a key driver in the initiation and progression of atherosclerotic plaques [6, 7].

We and others have shown that hypoglycaemia induces an acute and prolonged inflammatory response in people with type 1 diabetes and in people without diabetes on a cellular, functional and protein level [8, 9]. This inflammatory response includes an acute rise in neutrophil, lymphocyte and monocyte counts, with lymphocytes and monocytes remaining elevated for at least 7 days [8, 10]. In addition, hypoglycaemia acutely increases the secretion of proinflammatory cytokines by monocytes upon ex vivo stimulation. Lastly, hypoglycaemia evokes a range of circulating inflammatory proteins, many of which remain elevated for up to 7 days after the hypoglycaemic event [8].

The underlying mechanism driving this hypoglycaemia-induced inflammatory response is currently unclear. There are indications that adrenaline (epinephrine), which is part of the acute counterregulatory hormone response to hypoglycaemia, is involved [8, 11]. Studies dating back to the 1990s have shown that adrenaline elicits an acute rise (within minutes after administration) in leukocytes [12–14]. In the hyperinsulinaemic–hypoglycaemic clamp studies that our group has performed, we observed a consistent positive correlation between the adrenaline response to hypoglycaemia and the increase in granulocyte, lymphocyte and monocyte counts [8, 15]. We thus hypothesise that adrenaline is the main factor driving hypoglycaemia-induced inflammatory responses. To test this hypothesis, we investigated the acute and prolonged effects of adrenaline administration, using the same 1 h timeframe and target adrenaline levels as our previous clamp studies, on white blood cell counts, inflammatory cell function and circulating inflammatory markers in people with type 1 diabetes and without diabetes.

## Methods

### Population

This study was performed at the Radboud University Medical Center (Nijmegen, the Netherlands), approved by the local institutional review board (ClinicalTrials.gov registration no. NCT05990933) and performed according to the principles of the Declaration of Helsinki. All participants gave written informed consent. From September 2023 to September 2024, we recruited 15 participants with type 1 diabetes and 15 matched control participants without diabetes, matched for age ±3 years, gender ±1 participant and BMI ±2 kg/m^2^, through outpatient diabetes clinics, diabetes societies, advertisements and social media. Participants were eligible when they were aged between 18 and 75 years, had a BMI of 19–30 kg/m^2^ and were in general good health. The main exclusion criteria were an HbA_1c_ over 100 mmol/mol (11.3%) (for people with type 1 diabetes) or over 48 mmol/mol (for control participants), a cardiovascular event in the past 5 years (e.g. myocardial infarction), serious arrhythmias or conduction abnormalities on a standard electrocardiogram (for the full list see electronic supplementary material [ESM] Table 1). During the screening they were asked to fill in the modified Clarke score [16] to assess their awareness of hypoglycaemia. We did not collect race or ethnicity data, as we did not consider it relevant to the pathophysiological mechanisms under investigation. We aimed for an equal distribution of gender across both groups. Gender was determined by the status collected from the medical record which records gender as registered with the registry office.

### Experimental design

**Fig. 1:**
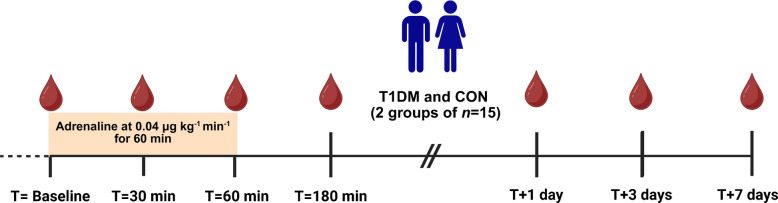
Study protocol. Adrenaline administration. CON, matched control participants without diabetes; T, timepoint; T1DM, participants with type 1 diabetes mellitus

On the day of the experiment (Fig. 1), participants came to the research facility in a fasting condition at 08:00 hours, having abstained from alcohol and caffeine- or nicotine-containing substances for at least 24 h, and from strenuous exercise for 48 h. Participants with type 1 diabetes were instructed to skip their morning insulin dose on the day of the intervention and adjust their basal insulin to avoid hypoglycaemia 24 h prior to the intervention. If participants with type 1 diabetes had a glucose higher than 10 mmol/l before the start of adrenaline, insulin was administered intravenously to correct it to just below 10 mmol/l, using an individualised correction bolus. We inserted peripheral intravenous catheters in the antecubital veins of each arm. The non-dominant arm was used for adrenaline administration and the dominant arm for blood sampling. After 30 min of equilibration following insertion of the cannulae, blood was drawn for baseline measurements. Adrenaline was then administered continuously for 60 min at a rate of 0.04 µg kg^−1^ min^−1^. During and up to 120 min after adrenaline administration, blood pressure and heart rate were continuously measured. At baseline and after 30 min, 60 min and 180 min, blood was sampled for measurement of insulin, catecholamines (adrenaline and noradrenaline [norepinephrine]) and inflammatory parameters. Blood was also obtained after 1, 3 and 7 days for measurement of inflammatory parameters.

### Serum and plasma measurements

Serum creatinine was determined with an enzymatic assay on a Cobas 8000 using module c702. Insulin levels were measured using a radioimmunoassay module E801 (Roche Diagnostics). HbA_1c_ was assessed by HPLC using a cation exchange column on a TOSOH G11 analyser according to the manufacturer instructions (Tosoh Bioscience). Plasma adrenaline and noradrenaline were assessed by an ultra-performance LC-MS/MS method. Plasma high-sensitivity C-reactive protein (hs-CRP) was quantified by ELISA (R&D Duoset ELISA Systems). The presence of 92 circulating inflammatory proteins was measured in EDTA plasma using a proximity extension assay (Olink Proteomics Inflammation Panel, Uppsala, Sweden). Quality control of the samples was performed after measurement according to Olink Proteomics instructions [17]. Two samples that did not pass quality control in the proximity extension assay were excluded from statistical analysis. Two additional samples were excluded from analysis due to missing data, resulting in four excluded samples in total. Overall, 74 of the 92 proteins (80%) were detected in at least 75% of the plasma samples.

### Isolation of peripheral blood mononuclear cells and monocytes

Peripheral blood mononuclear cells (PBMCs) were isolated from whole blood in Sepmate tubes (Stemcell Technologies) using density centrifugation over Ficoll-Paque (GE Healthcare). Monocytes were separated from PBMCs by CD14-negative selection using indirect magnetic labelling (Miltenyi Biotec). Cell composition of whole blood and purity of monocytes were assessed using a Sysmex XN-450 Hematology Analyzer (Sysmex Corp., Kobe, Japan), which uses fluorescence flow cytometry to differentiate between immune cell subsets.

### Monocyte stimulation

Monocytes were seeded in flat-bottom 96-well plates (100,000 cells/well) and cultured with RPMI medium supplemented with 10% human pooled serum, 10 μg/ml Pam3Cys (P3C) or 10 ng/ml lipopolysaccharide (LPS) from *Escherichia coli* for 24 h. Supernatants were collected and stored at −20°C until cytokines were measured. IL-6, IL-1β and TNF production was quantified by commercially available ELISA kits (R&D Duoset ELISA Systems).

### Statistical analysis

Statistical analyses were performed using SPSS Statistics for Windows (version 29.0, IBM) R (version 4.4.1) [18]. A sample size of 15 per group was required to show a significant increase of monocytes following adrenaline administration, using the hypoglycaemia-induced increase in monocyte count as a reference [8], with a power of 90% and a two-sided significance level of 0.05 to detect an effect size of 0.167 ± 0.036 × 10^9^/l (*n*=47). All normally distributed data are shown as mean ± SEM, unless otherwise indicated. Independent Student’s *t* tests were used to compare continuous data that were normally distributed, whereas the Mann–Whitney *U* test was used for data that were not normally distributed. Consecutive changes in cell counts were analysed by mixed model analyses. Data from the hypoglycaemic clamp study by Verhulst et al [8], including 47 participants with type 1 diabetes, were used for visual comparisons only; no statistical tests were performed between this group and the adrenaline groups. The α was set at 0.05 throughout. The proximity extension assay data were analysed and visualised using the R programming language and R package ‘ggplot2’ [19]. To determine significant changes in inflammatory proteins from baseline to other timepoints, Wilcoxon matched-pairs tests were performed. Differences in proteins between type 1 diabetes and control participants at baseline were assessed using a Wilcoxon rank sum test.

## Results

### Baseline characteristics

The characteristics of the two groups can be found in Table 1. Although adrenaline levels at baseline were lower in participants with type 1 diabetes compared with control participants (0.11 ± 0.02 vs 0.23 ± 0.03 nmol/l, *p*=0.005), they peaked at 30 min to similar levels of 2.37 ± 0.28 nmol/l and 2.23 ± 0.25 nmol/l in participants with type 1 diabetes and control participants, respectively, and remained stable at 60 min at 2.12 ± 0.31 nmol/l and 2.24 ± 0.28 nmol/l, without significance between the groups. After 180 min, adrenaline levels returned to baseline values (ESM Fig. 1). Noradrenaline levels rose slightly, peaking at 30 min and returning back to baseline after 180 min (ESM Fig. 2). Continuous glucose monitoring did not show abnormal blood glucose excursions in participants with type 1 diabetes after the adrenaline administration. Lastly, the type 1 diabetes group included five people with impaired awareness of hypoglycaemia; we did not observe any differences in the inflammatory results between participants with impaired vs normal awareness of hypoglycaemia.

**Table 1:** Baseline characteristics of the participants receiving adrenaline administration

Characteristic	T1DM	CON	*p* value
*n*	15	15	
Male/female	8/7	8/7	1.000
Age (years)	44.3 ± 15.9	43.5 ± 20.7	0.819
BMI (kg/m^2^)	24.7 ± 2.7	23.6 ± 2.5	0.250
eGFR (ml/min per 1.73 m^2^)	100.6 ± 11.3	96.9 ± 19.1	0.141
HbA_1c_ (mmol/mol)	58.1 ± 9.8	34.9 ± 3.9	<0.001*
HbA_1c_ (%)	7.5 ± 3	5.3 ± 2.5	<0.001*
Diabetes duration (years)	20.5 ± 11.9	–	
Insulin therapy			
MDI	40.0 (*n*=6)	–	
CSII	60.0 (*n*=9)	–	
Insulin dose (IU/day)	39.1 ± 17.7	–	

Data are presented as number (%) or mean ± SD. Shapiro–Wilk test was used to assess normality. Comparisons between the two groups were done using the unpaired *t* test for normally distributed data and Mann–Whitney *U* test for non-normally distributed data. Two-sided *p* values are presented

^*^p<0.001 vs T1DM

CON, matched control participants without diabetes; CSII, continuous subcutaneous insulin infusion; eGFR, eGFR as calculated with the Chronic Kidney Disease Epidemiology Collaboration (CKD-EPI) formula; MDI, multiple daily injections; T1DM, participants with type 1 diabetes mellitus

### Circulating immune cell response

**Fig. 2:**
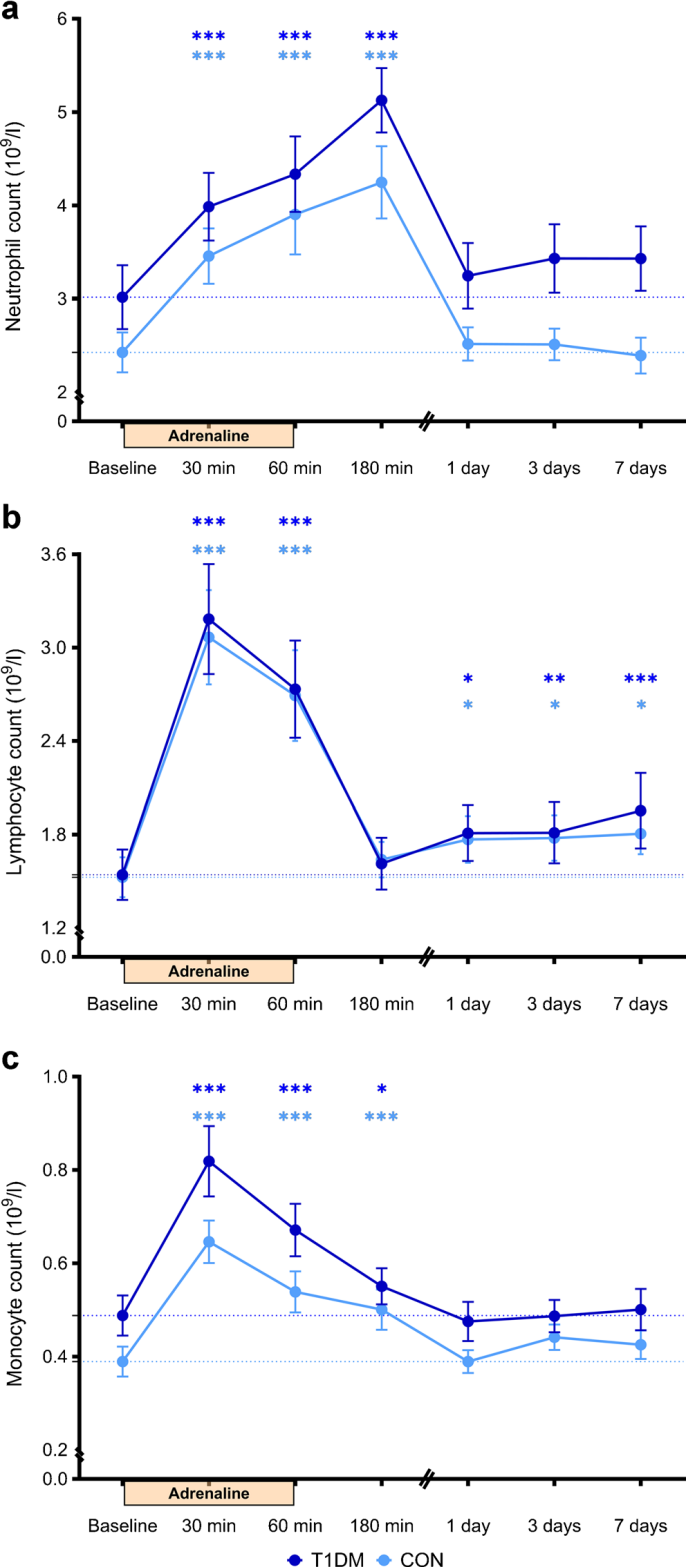
Circulating immune cell counts after adrenaline administration. Neutrophil count (**a**), lymphocyte count (**b**) and monocyte count (**c**) for participants with type 1 diabetes mellitus (T1DM; dark blue, *n*=15) and matched control participants without diabetes (CON; light blue, *n*=15). Data are presented as mean ± SEM; *p<0.05, **p<0.01 and ***p<0.001 vs baseline, based on mixed model analysis

Absolute neutrophil, lymphocyte and monocyte counts acutely increased in both groups in response to adrenaline administration (Fig. 2a–c). Lymphocytes and monocytes peaked after 30 min, while neutrophils showed a protracted response, peaking after 180 min. No significant differences were observed between the groups in any of the counts, neither at baseline nor in response to adrenaline. Neutrophils and monocytes returned to baseline after 1 day, whereas lymphocytes remained elevated for the full 7 days in both groups.

### Cytokine production of ex vivo stimulated monocytes

**Fig. 3:**
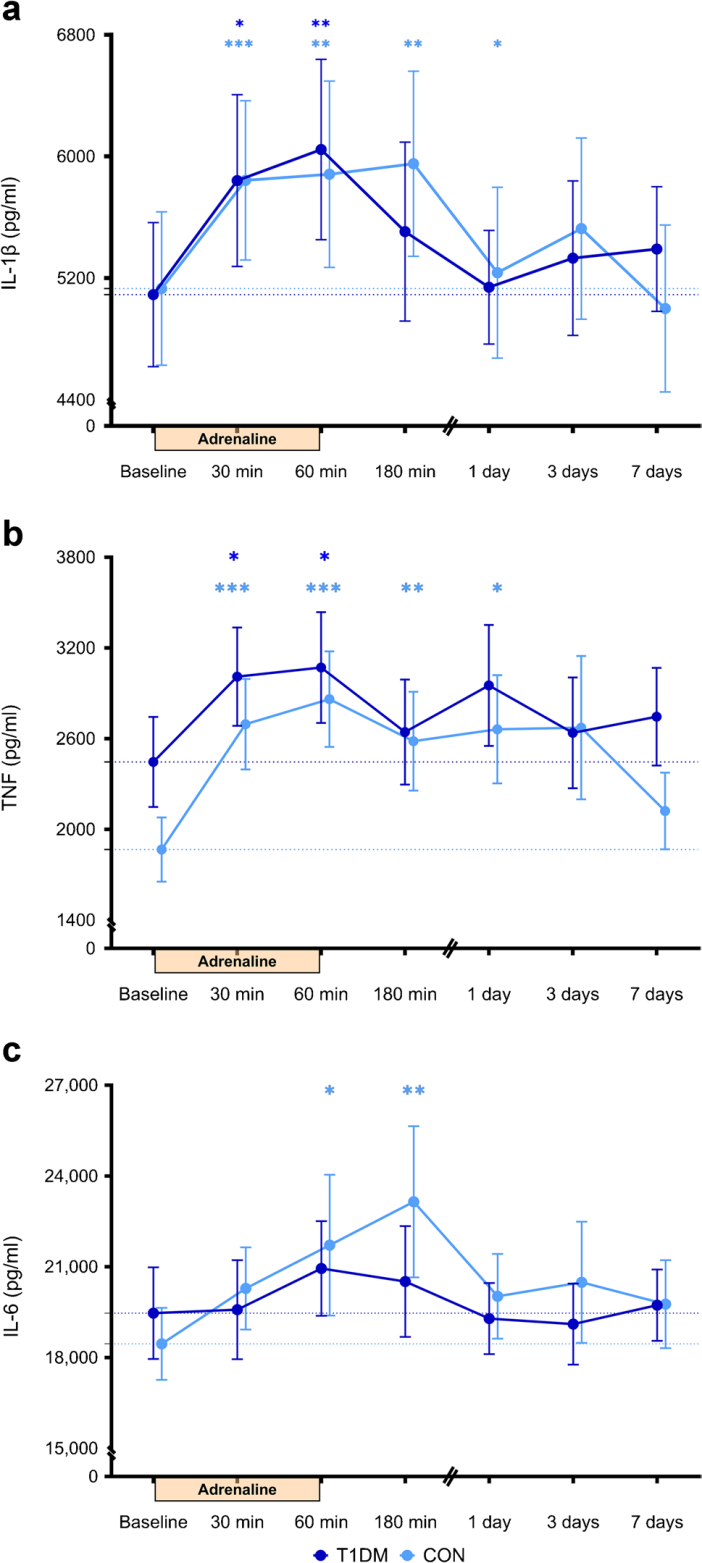
Ex vivo cytokine production by monocytes upon LPS stimulation after adrenaline administration. IL-1β (**a**), TNF (**b**) and IL-6 (**c**) upon LPS stimulation, for participants with type 1 diabetes mellitus (T1DM; dark blue, *n*=15) and matched control participants without diabetes (CON; light blue, *n*=15). Data are presented as mean ± SEM; *p<0.05, **p<0.01 and ***p<0.001 vs baseline based on mixed model analysis

At baseline, no significant differences were observed between the two groups in the secretion of IL-1β, IL-6 and TNF upon ex vivo LPS and P3C stimulation of monocytes (Fig. 3a–c, ESM Fig. 3a–c). Adrenaline significantly increased IL-1β and TNF production upon LPS stimulation after 30 and 60 min, without differences between the groups (Fig. 3a, b). Adrenaline did not affect IL-6 production after LPS stimulation in participants with type 1 diabetes, while it significantly increased IL-6 production in control participants, peaking after 180 min (Fig. 3c). Similar patterns in cytokine production were observed when monocytes were stimulated with P3C, although the effect was less pronounced compared with LPS stimulation (ESM Fig. 3a–c).

### Analysis of 92 circulating inflammatory proteins

**Fig. 4:**
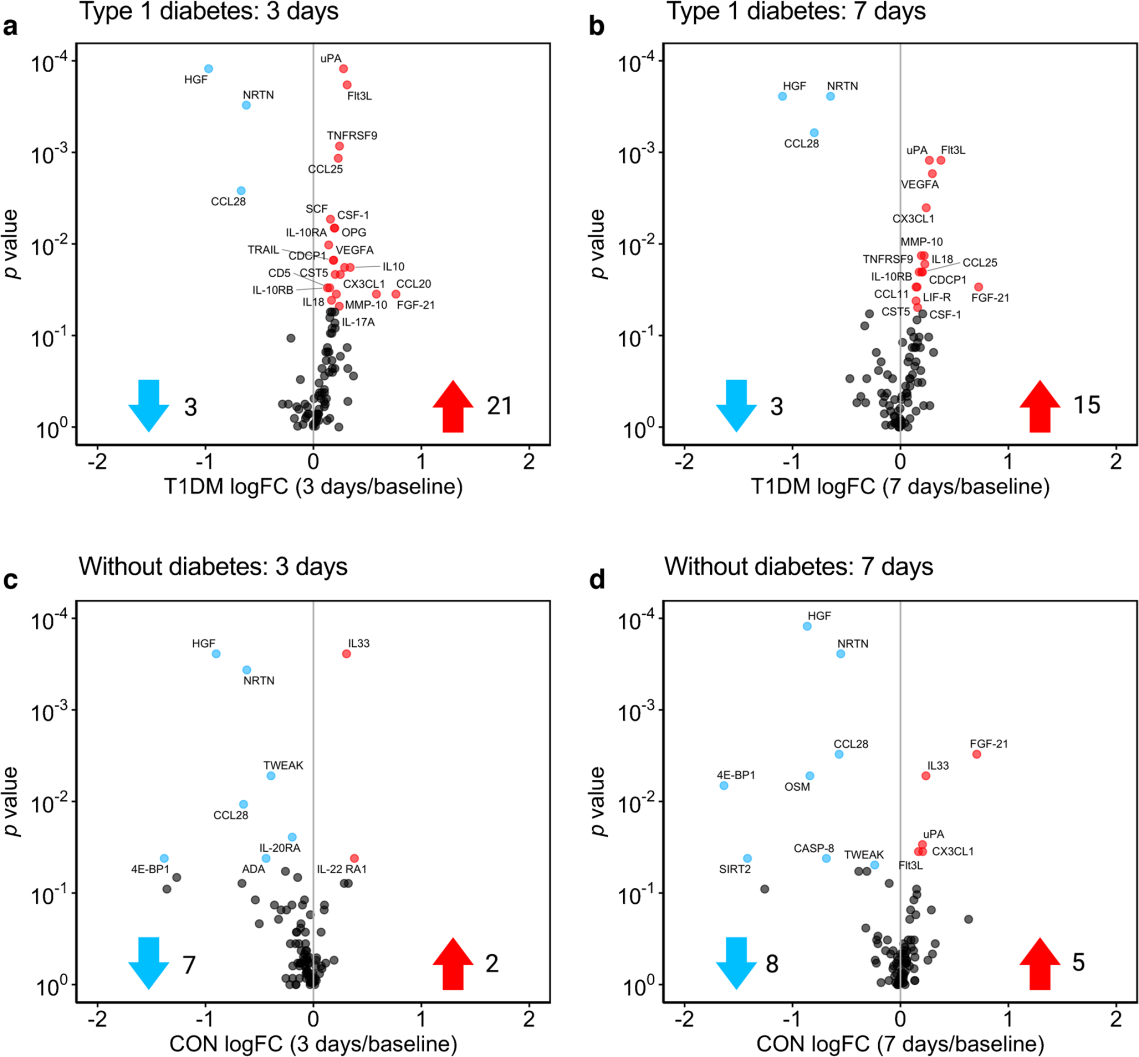
(**a**–**d**) Volcano plots of circulating inflammatory proteins per group (participants with type 1 diabetes mellitus [T1DM; *n*=15], matched control participants without diabetes [CON; *n*=15]) and timepoint compared with baseline. Dots in blue represent significantly decreased proteins and dots in red represent significantly increased proteins (Wilcoxon paired test, unadjusted *p* value <0.05). The blue and red arrows represent the number of significantly decreased (blue) and increased (red) proteins. FC, fold change

To gain a more detailed understanding of the inflammatory response, we performed a proteomics analysis of 92 circulating inflammatory proteins after adrenaline administration. At baseline, nine proteins were significantly different between both groups, with seven proteins lower and two higher in participants with type 1 diabetes compared with control participants (ESM Fig. 4a). Adrenaline administration resulted in an increase in circulating levels of IL-6, IL-10 and oncostatin-M after 60 min in both groups (ESM Fig. 4b, d). After 1 day, only chemokine (C-X3-C motif) ligand 1 (CX3CL1) was elevated in type 1 diabetes while IL-6, CX3CL1 and urokinase-type plasminogen activator (uPA) were elevated in control participants (ESM Fig. 4c, e). Interestingly, an elevation of 21 proteins was observed in participants with type 1 diabetes after 3 days, while this was true for only two proteins in control participants (Fig. 4a, c). Of the 21 inflammatory proteins that were increased after 3 days in type 1 diabetes, 13 remained elevated at day 7 along with two additional proteins, resulting in a total of 15 significantly increased proteins at this timepoint (Fig. 4b). In control participants, only five proteins were significantly increased at day 7, of which one (IL-33) was also elevated at day 3 (Fig. 4d, ESM Fig. 4e). Proteins that were elevated at day 7 in both groups were uPA, Fms-like tyrosine kinase 3 ligand (Flt3L), CX3CL1 and fibroblast growth factor 21 (FGF-21) (Fig. 4b, d). For a complete overview of the *p* values for each protein see ESM Tables 2–10.

### hs-CRP

**Fig. 5:**
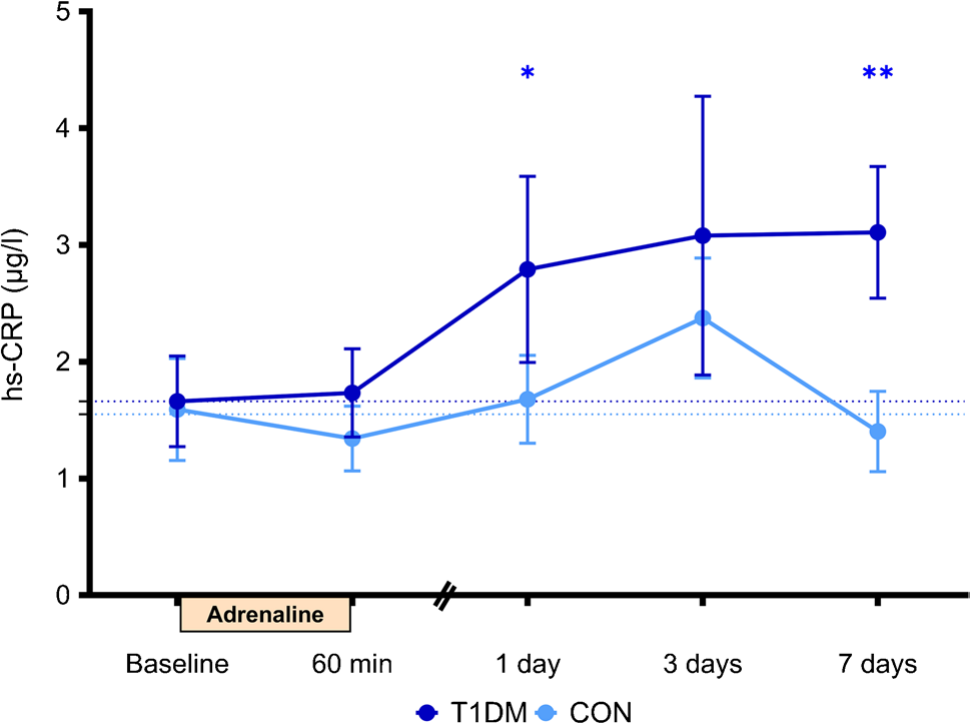
Circulating inflammatory protein hs-CRP after adrenaline administration for participants with type 1 diabetes mellitus (T1DM; dark blue, *n*=15) and matched control participants without diabetes (CON; light blue, *n*=15). Data are presented as mean ± SEM; *p<0.05 and **p<0.01 vs baseline based on mixed model analysis

hs-CRP levels did not differ between the groups at baseline. In participants with type 1 diabetes, but not in control participants, hs-CRP increased significantly 1 day after adrenaline administration and continued to rise until day 7 (Fig. 5), resulting in a significant difference between the groups at day 7 (*p*=0.011).

### Adrenaline administration vs hypoglycaemia

When comparing our findings with the earlier results obtained in 47 participants with type 1 diabetes who underwent a hypoglycaemic clamp [8], we observed similar inflammatory responses. The acute increase in cell counts (ESM Fig. 5a–c) after adrenaline followed an analogous pattern to that observed in response to hypoglycaemia. The similarities continued with the sustained increase in lymphocytes after hypoglycaemia and adrenaline (ESM Fig. 5b). Additionally, cytokine production upon stimulation of monocytes with LPS (ESM Fig. 6a–c) and P3C (ESM Fig. 7a–c) after adrenaline administration showed a comparable pattern to that following hypoglycaemia. Adrenaline administration and hypoglycaemia elicited a similar pattern in hs-CRP, with both interventions significantly increasing hs-CRP after 1 day in type 1 diabetes (ESM Fig. 8). Finally, the proteomic analysis also identified overlapping protein profiles in the type 1 diabetes groups exposed to adrenaline administration or hypoglycaemia after 7 days. Both interventions significantly altered the same ten inflammatory proteins, including uPA, CX3CL1 and vascular endothelial growth factor A (VEGFA) (ESM Fig. 9).

## Discussion

With this study we show that adrenaline administration elicits both an acute and a sustained inflammatory response on a cellular, functional and protein level, both in people with type 1 diabetes and in those without diabetes. Acutely, this response is characterised by a rise in white blood cell counts and a shift towards a more proinflammatory monocyte phenotype, illustrated by enhanced ex vivo production of proinflammatory cytokines. The prolonged inflammatory response induced by adrenaline is reflected by persistently elevated lymphocyte counts for up to 7 days in both groups, along with increased levels of several proinflammatory proteins, including uPA, Flt3L, CX3CL1 and FGF-21. When comparing these data with the inflammatory effects of hypoglycaemia, as reported earlier, a considerable amount, albeit not all, of the effects overlapped. These findings support a prominent role of adrenaline in driving inflammatory responses following hypoglycaemia.

Our data investigating the effects of adrenaline on immune cells are in line with previous literature, which has primarily focused on the short-term effect of adrenaline on absolute white blood cell counts, showing a rapid increase in white blood cells following adrenaline administration in people without diabetes [14, 20, 21]. We extend these earlier published data by several new observations. First, we show that this cellular response includes all leukocyte cell types and does not differ between people with type 1 diabetes or without diabetes. Second, we demonstrate that the effect on lymphocytes is sustained for at least 7 days. Third, we found that adrenaline affects various other components of the inflammatory response complex, including the production of cytokines and the release of inflammatory proteins, which is not limited to an acute response.

Despite the similar patterns between hypoglycaemia and adrenaline administration, there are also several differences between the two interventions. First, monocytes returned to baseline 1 day after adrenaline administration, whereas they remained elevated for 7 days after hypoglycaemia (ESM Fig. 5c). Second, hypoglycaemia triggered a stronger inflammatory protein response than adrenaline alone, with a greater number of proteins remaining significantly elevated in both groups. Notably, the counterregulatory hormone response to hypoglycaemia involves more than just adrenaline. In particular, elevations in plasma cortisol may have contributed to the inflammatory response following hypoglycaemia, since this hormone promotes migration of monocytes and neutrophils from the bone marrow [22] and has been shown to induce a chronic inflammatory state when persistently elevated [23].

The adrenaline response to stressors can be traced back to the classical fight or flight response. Indeed, just as adrenaline activates the cardiovascular and musculoskeletal systems in anticipation of a fight or flight response, it also prepares the immune system for incoming vital threats, such as hypoglycaemia [24]. While activation of the immune system during threatening situations is evolutionarily advantageous, repeated activation, triggered by recurrent hypoglycaemic events, may lead to immune dysregulation and harmful effects.

The rapid increase in white blood cells in response to adrenaline can be explained by a mechanism known as demargination, i.e. mobilisation of immune cells from marginal pools, such as blood vessels, bone marrow, lymph nodes and lungs. Demargination is mainly mediated by β_2_-adrenoceptors, which are expressed by all white blood cells [11], but α-adrenoreceptors also play a role in the recruitment of, in particular, lymphocytes, as demonstrated by α-blockade during a hypoglycaemic clamp [25]. The increase in proinflammatory cytokine production by monocytes upon LPS stimulation after adrenaline administration is probably an indirect effect of adrenaline. In fact, literature suggests that ex vivo β-adrenoreceptor activation in human monocytes primarily exerts anti-inflammatory and immunosuppressive effects, rather than a proinflammatory response [26, 27]. Since the demargination process mainly involves recruitment of non-classical monocytes, which are known to produce higher levels of proinflammatory cytokines upon ex vivo stimulation than classical monocytes [11, 28], the rise in proinflammatory cytokine production probably resulted from a shift in monocyte subsets.

Apart from the acute response, we observed an increase in absolute lymphocyte count for at least 7 days, both in people with type 1 diabetes and in those without diabetes. This finding may be attributed to an increase in natural killer cells, which have a particularly strong affinity for catecholamines [13, 14] and a lifespan of approximately 2 weeks [29]. While inflammatory proteins were elevated in both groups after 7 days, participants with type 1 diabetes showed a more robust response compared with control participants. The stronger effect of adrenaline in type 1 diabetes may be due to increased sensitivity of the inflammatory response to adrenaline. Interestingly, we previously reported that recurrent hypoglycaemic events reduce adrenaline responses to a subsequent event, but do not change the inflammatory response [30], possibly due to an upregulation of adrenergic receptors [31]. Alternatively, decreased degradation of adrenaline due to lesser activity of monoamine oxidase (MAO) or catecholamine O-methyltransferase (COMT) may also explain the stronger response in type 1 diabetes [32].

The sustained inflammatory effects of adrenaline administration are further supported by the prolonged increase in inflammatory proteins and in hs-CRP, a well-known marker for chronic and subclinical inflammation that is associated with the development of atherosclerosis [33]. In theory, these sustained effects pose a serious cardiovascular risk for people with diabetes who experience hypoglycaemia, given the well-established link between inflammation and atherosclerosis development [34], independent of classical risk factors [35]. This cardiovascular risk is supported by the elevation of VEGFA, an atherogenic biomarker [36] observed only in type 1 diabetes after 7 days, which is known to be released through β1- and β2-adrenoreceptor activation [37, 38].

Both groups showed elevated levels of uPA, Flt3L, FGF-21 and CX3CL1 after 7 days. These proteins were also elevated in previous hypoglycaemic clamp studies, suggesting a direct role of adrenaline in increasing these circulating proteins. This elevated production could be driven by activation of β-adrenergic receptors, a subset of G protein-coupled receptors, leading to increased intracellular cAMP, and consequently enhancing the production of inflammatory proteins by e.g. leukocytes, the liver or endothelium [12]. In the context of cardiovascular disease, CX3CL1 and uPA are of particular interest, since both proteins have been associated with the development of atherosclerosis [39–41]. uPA is a mediator of fibrinolysis and is highly expressed by cells in advanced atherosclerotic plaques. Adrenaline has been shown to play a role in thrombosis and is associated with the formation of denser platelet–fibrin clots and decreased rate of fibrinolysis [42]. Elevated uPA might therefore be a compensatory mechanism to promote fibrinolysis despite increased density of the clots. CX3CL1 has been identified as a demargination marker on cytotoxic effector leukocyte subsets and is believed to mediate attachment of cells to the endothelium. In addition, CX3CL1 has been shown to play a role in the pathogenesis of plaque vulnerability [40].

Our study has several strengths. First, we were able to mimic the adrenaline levels as commonly achieved during hypoglycaemia, at least under clamped conditions (ESM Table 1). This allowed us to study the long-term effect of adrenaline on the immune system in vivo. Second, by assessing cell counts, cell function and circulating proteins, our study provides a multidimensional analysis of the impact of adrenaline on the inflammatory architecture at a more detailed scale than previously achieved. Third, the two groups of participants were well matched, suggesting that differences between the groups were the consequence of diabetes or associated factors (e.g. prior hypoglycaemia). Lastly, our results showed no significant differences in the main outcomes (leukocytes, adrenaline levels) between genders. This, taken together with the equal gender distribution, supports the applicability of our findings to the overall population. Our study also has limitations. First, we have no information about the origin of the cells released in response to adrenaline, since we did not perform bone marrow aspirations. Hence, we cannot determine whether the cells are derived from de novo production or from demargination or both. Second, our study was open label, which may have introduced bias as the participants may have been subconsciously in a slight state of stress while anticipating the effects of adrenaline.

In conclusion, this study shows that adrenaline evokes both an acute and a sustained inflammatory response in people with type 1 diabetes and in those without diabetes, on a cellular, functional and protein level. While the acute inflammatory effects of adrenaline largely overlapped with the inflammatory response to (experimental) hypoglycaemia, there were also differences, particularly in relation to the sustained effects. Our findings strongly suggest that adrenaline drives the acute proinflammatory response to hypoglycaemia, while also contributing significantly to the sustained inflammatory response. Future studies are needed to reveal the molecular mechanisms involved in adrenaline’s inflammatory effect, especially studies eliminating adrenaline from the equation.

## Supplementary Information

Below is the link to the electronic supplementary material.

ESM (PDF 1.72 MB)

## Data Availability

Data supporting the findings of this study are available from the corresponding author upon reasonable request.

## Abbreviations

CX3CL1: Chemokine (C-X3-C motif) ligand 1
FGF-21: Fibroblast growth factor 21
Flt3L: Fms-like tyrosine kinase 3 ligand
hs-CRP: High-sensitivity C-reactive protein
LPS: Lipopolysaccharide
PBMC: Peripheral blood mononuclear cell
P3C: Pam3Cys
uPA: Urokinase-type plasminogen activator
VEGFA: Vascular endothelial growth factor A
