# Supplementary material for: Adrenaline is a prominent driver of inflammatory responses following hypoglycaemia
Source: Diabetologia. 2026 Jan 29;69(6):1675–85. doi: 10.1007/s00125-026-06667-9 (PMC13109272; doi:10.1007/s00125-026-06667-9)
Supplement: Supplementary file 1 — ESM (PDF 1.72 MB) [file 125_2026_6667_MOESM1_ESM.pdf]

ELECTRONIC SUPPLEMENTARY MATERIAL (ESM) - Adrenaline is a prominent driver of inflammatory responses following hypoglycaemia

Ilyas F. Mustafajev[1*], Marijn S. Hendriksz[1*], Rinke Stienstra[1,4], Cees J., Tack[1], Bastiaan E. de Galan[1,2,3,†], Rick I. Meijer[1,†]

1. *Department of Internal Medicine, Radboud University Medical Center, Nijmegen, the Netherlands.*
2. *Department of Internal Medicine, Maastricht University Medical Centre, MUMC+, Maastricht, The Netherlands*
3. *CARIM Cardiovascular Research Institute Maastricht, Maastricht University, Maastricht, The Netherlands*
4. *Division of Human Nutrition and Health, Wageningen University, Wageningen, The Netherlands*

* Shared first authorship

† Shared last authorship

ESM Table 1: Inclusion and exclusion criteria

**Inclusion criteria**
Ability to provide written informed consent
Body-Mass Index: 19-30 kg/m2
Age ≥16 years, ≤ 75 years
Blood pressure: <140/90 mmHg
Non-smoking
Electrocardiogram not showing any serious arrythmias (PVCs and PACs accepted)
**Participants with diabetes specific criteria**
Insulin treatment according to basal-bolus insulin regimen (injections or insulin pump)
Duration of diabetes > 1 year
HbA1c < 100 mmol/mol
HbA1c < 11.3%
**Exclusion criteria**
Any event of cardiovascular disease in the past 5 years (e.g. myocardial infarction, stroke, symptomatic peripheral arterial disease)
Pregnancy or breastfeeding or unwillingness to undertake measures for birth control
Epilepsy
Current treatment with Alpha- or beta-blockers (e.g. doxazosin, propranolol)
History of panic disorders
History of Arrhythmias
Use of immune-modifying drugs or antibiotics
Use of tricyclic antidepressants or MAO inhibitors

Use of statins with the inability to stop statins >2 weeks before the investigational day
Any infection with systemic symptoms in the 2 weeks before the investigational day
Previous vaccination in the 2 weeks before the investigational day
Proliferative retinopathy
Nephropathy with an estimated glomerular filtration rate (by MDRD) <60ml/min/1.73m2

ESM Table 2: Results of Wilcoxon rank-sum test comparing inflammatory protein levels between participants with type 1 diabetes mellitus (T1DM, n=15) and matched controls without diabetes mellitus (CON, n=15) at baseline. Differences were calculated as T1DM minus CON. See ESM Table 11 for definitions of OLINK protein abbreviations.

Proteins	P value	FDR	Difference	Fold change	FClog	plog
**CCL23**	0,00395584	0,30213041	-0,42764000	0,74347699	-0,42764000	0,51980556
**MMP-10**	0,01983813	0,46204583	-0,61192867	0,65432139	-0,61192867	0,33531494
**CD8A**	0,02214239	0,39250363	-0,47213800	0,72089548	-0,47213800	0,40615632
**EN-RAGE**	0,02459655	0,30213041	-0,53474400	0,69028115	-0,53474400	0,51980556
**4E-BP1**	0,03338617	0,46204583	-1,07200200	0,47565848	-1,07200200	0,33531494
**CSF-1**	0,03641884	0,46204583	-0,13866333	0,90836037	-0,13866333	0,33531494
**IL-22 RA1**	0,04030848	0,50593182	0,52357467	1,43751266	0,52357467	0,29590800
**IL-10RA**	0,04676907	0,50593182	-0,49093800	0,71156231	-0,49093800	0,29590800
**IL4**	0,04798026	0,46204583	0,57932067	1,49414552	0,57932067	0,33531494
**DNER**	0,05005914	0,50593182	-0,17635400	0,88493660	-0,17635400	0,29590800
**TSLP**	0,05032799	0,46204583	0,52243400	1,43637654	0,52243400	0,33531494
**MCP-1**	0,05074689	0,46204583	-0,28223933	0,82231364	-0,28223933	0,33531494
**SLAMF1**	0,07553097	0,50593182	0,26072200	1,19807814	0,26072200	0,29590800
**IL-10RB**	0,08303098	0,50593182	-0,15168467	0,90019867	-0,15168467	0,29590800
**TGF-alpha**	0,10279553	0,46204583	-0,20176067	0,86948879	-0,20176067	0,33531494
**IL8**	0,10520405	0,50593182	-0,28968933	0,81807820	-0,28968933	0,29590800
**SCF**	0,12234586	0,50593182	-0,16628067	0,89113711	-0,16628067	0,29590800
**LIF**	0,13654780	0,60457002	0,18595333	1,13756843	0,18595333	0,21855339
**Flt3L**	0,13659896	0,50593182	-0,22660400	0,85464430	-0,22660400	0,29590800
**ADA**	0,13974782	0,67526970	-0,29029133	0,81773691	-0,29029133	0,17052274
**TRAIL**	0,15272235	0,60457002	-0,16917600	0,88935049	-0,16917600	0,21855339
**CCL4**	0,17311970	0,60457002	0,23879267	1,18000475	0,23879267	0,21855339
**VEGFA**	0,18306863	0,50593182	-0,17089933	0,88828878	-0,17089933	0,29590800
**IL-18R1**	0,18906820	0,50593182	0,17654000	1,13017016	0,17654000	0,29590800
**IL33**	0,21050926	0,59409286	0,15233467	1,11136651	0,15233467	0,22614567
**IL-20**	0,22514627	0,68258128	-0,24227333	0,84541210	-0,24227333	0,16584562
**CASP-8**	0,23143822	0,50593182	-0,42783333	0,74337737	-0,42783333	0,29590800
**SIRT2**	0,23698242	0,59409286	-0,78097133	0,58197483	-0,78097133	0,22614567
**CXCL5**	0,24286941	0,50593182	0,66417400	1,58466073	0,66417400	0,29590800
**FGF-5**	0,24857987	0,50593182	0,10354667	1,07441151	0,10354667	0,29590800
**CST5**	0,26067623	0,60457002	-0,21069933	0,86411826	-0,21069933	0,21855339
**CXCL10**	0,26631047	0,82323214	0,32704067	1,25443756	0,32704067	0,08447768
**uPA**	0,27183229	0,75736095	-0,12048667	0,91987729	-0,12048667	0,12069709
**STAMBP**	0,28872927	0,68258128	-0,68570133	0,62170353	-0,68570133	0,16584562
**Beta-NGF**	0,29869828	0,50593182	-0,05072333	0,96545215	-0,05072333	0,29590800
**IL-15RA**	0,29945601	0,81457955	-0,10093067	0,93243130	-0,10093067	0,08906650

**CCL28**	0,31661987	0,50593182	0,30200400	1,23285574	0,30200400	0,29590800
**CCL19**	0,31835619	0,94050285	-0,18411067	0,88019149	-0,18411067	0,02663989
**IL-17C**	0,31889396	0,82323214	-0,29044333	0,81765076	-0,29044333	0,08447768
**IL10**	0,32490889	0,96341179	0,18299067	1,13523476	0,18299067	0,01618804
**CXCL1**	0,35221268	0,50593182	0,44301667	1,35944395	0,44301667	0,29590800
**NRTN**	0,36598171	0,94050285	0,22336133	1,16745046	0,22336133	0,02663989
**TNF**	0,37041357	0,95339750	0,23706933	1,17859605	0,23706933	0,02072599
**IL-17A**	0,38188884	0,95339750	-0,27822067	0,82460741	-0,27822067	0,02072599
**IL6**	0,38867407	0,94050285	0,22114733	1,16566023	0,22114733	0,02663989
**GDNF**	0,41743934	0,90230223	-0,12553200	0,91666596	-0,12553200	0,04464797
**TRANCE**	0,41769645	0,94050285	-0,21155467	0,86360610	-0,21155467	0,02663989
**CCL3**	0,41920134	0,94050285	0,15862267	1,11622098	0,15862267	0,02663989
**OSM**	0,42343893	0,89196950	-0,24835267	0,84185714	-0,24835267	0,04965000
**CD40**	0,48485334	0,93219913	0,24432467	1,18453815	0,24432467	0,03049131
**CXCL6**	0,50887687	0,96341179	-0,18123400	0,88194830	-0,18123400	0,01618804
**ST1A1**	0,51512844	0,90230223	0,32795867	1,25523603	0,32795867	0,04464797
**FGF-21**	0,55162650	0,94050285	-0,19357200	0,87443800	-0,19357200	0,02663989
**IL5**	0,55690753	0,97605569	0,22253933	1,16678548	0,22253933	0,01052540
**IL7**	0,56215018	0,83277822	-0,12605533	0,91633350	-0,12605533	0,07947064
**HGF**	0,63234845	0,96341179	0,13158200	1,09549432	0,13158200	0,01618804
**CXCL9**	0,63260914	0,96341179	0,15149067	1,11071653	0,15149067	0,01618804
**MCP-3**	0,63403942	0,96341179	-0,06993600	0,95268026	-0,06993600	0,01618804
**CCL11**	0,65172503	0,83277822	-0,07189400	0,95138818	-0,07189400	0,07947064
**IL13**	0,65261942	0,50593182	0,11680067	1,08432758	0,11680067	0,29590800
**CX3CL1**	0,66156327	0,96341179	-0,05345867	0,96362340	-0,05345867	0,01618804
**IL-24**	0,67473644	0,96341179	0,08977067	1,06420100	0,08977067	0,01618804
**MMP-1**	0,67585006	0,94050285	0,14643600	1,10683180	0,14643600	0,02663989
**MCP-2**	0,68535417	0,97631573	0,08213467	1,05858320	0,08213467	0,01040971
**IL-2RB**	0,69879722	0,89196950	-0,05183867	0,96470606	-0,05183867	0,04965000
**CDCP1**	0,69998223	0,94050285	0,09374067	1,06713350	0,09374067	0,02663989
**CXCL11**	0,70035952	0,81457955	0,12950667	1,09391957	0,12950667	0,08906650
**IL-20RA**	0,70062838	1,00000000	0,06013000	1,04255970	0,06013000	0,00000000
**TWEAK**	0,70119791	0,97605569	0,07480400	1,05321794	0,07480400	0,01052540
**FGF-23**	0,72657705	0,96341179	-0,05080333	0,96539862	-0,05080333	0,01618804
**TNFRSF9**	0,73698951	0,97678270	-0,05356400	0,96355305	-0,05356400	0,01020204
**IFN-gamma**	0,74185532	0,98861635	0,16400933	1,12039646	0,16400933	0,00497221
**OPG**	0,76055195	1,00000000	-0,04255533	0,97093368	-0,04255533	0,00000000
**ARTN**	0,76473679	0,94050285	-0,08790600	0,94088741	-0,08790600	0,02663989
**IL-12B**	0,80953168	0,96341179	-0,05346600	0,96361850	-0,05346600	0,01618804
**CCL20**	0,81040349	0,96341179	0,08081133	1,05761265	0,08081133	0,01618804
**AXIN1**	0,81113816	0,97678270	0,18636133	1,13789018	0,18636133	0,01020204
**CD244**	0,81284708	0,96341179	0,05829333	1,04123328	0,05829333	0,01618804
**IL2**	0,82263621	0,94050285	-0,02819333	0,98064758	-0,02819333	0,02663989
**CD5**	0,84456471	0,96341179	0,02558733	1,01789400	0,02558733	0,01618804

**PD-L1**	0,85274706	0,94050285	0,04236933	1,02980368	0,04236933	0,02663989
**MCP-4**	0,86313982	0,97678270	0,03650000	1,02562263	0,03650000	0,01020204
**TNFSF14**	0,86651913	1,00000000	0,05113733	1,03608139	0,05113733	0,00000000
**TNFB**	0,89689888	1,00000000	-0,01643200	0,98867482	-0,01643200	0,00000000
**NT-3**	0,90273453	0,97631573	0,01941267	1,01354677	0,01941267	0,01040971
**CCL25**	0,91755971	0,94050285	-0,02264200	0,98442827	-0,02264200	0,02663989
**IL18**	0,92903934	0,94050285	0,01590467	1,01108527	0,01590467	0,02663989
**CD6**	0,94060223	0,96341179	-0,01701533	0,98827515	-0,01701533	0,01618804
**LAP TGF-beta-1**	0,95036648	0,97631573	0,01132200	1,00787869	0,01132200	0,01040971
**LIF-R**	0,96950933	1,00000000	0,00403867	1,00280331	0,00403867	0,00000000
**FGF-19**	0,98571128	0,98861635	-0,00593067	0,99589761	-0,00593067	0,00497221
**IL-1 alpha**	0,99827375	0,96341179	0,00063533	1,00044048	0,00063533	0,01618804

ESM Table 3: Results of Wilcoxon matched pairs test comparing inflammatory protein levels between baseline (T0) and 60 minutes (T60) after adrenaline administration in participants with type 1 diabetes mellitus (T1DM, n=15). Differences were calculated as T60 minus T0. See ESM Table 11 for definitions of OLINK protein abbreviations.

Proteins	P value	FDR	Difference	Fold change	FClog	plog
**IL10**	0,00006104	0,00561523	1,54266000	2,91331157	1,54266000	2,25063211
**IL6**	0,00012207	0,00561523	0,94849933	1,92986420	0,94849933	2,25063211
**NRTN**	0,00335693	0,09826660	-0,34923067	0,78500260	-0,34923067	1,00759406
**CXCL10**	0,00427246	0,09826660	-0,31353067	0,80467010	-0,31353067	1,00759406
**Flt3L**	0,00537109	0,09882813	-0,20107067	0,86990474	-0,20107067	1,00511944
**OSM**	0,01025391	0,15722656	0,57704067	1,49178608	0,57704067	0,80347408
**IL-17C**	0,01507568	0,19813756	-0,21310133	0,86268075	-0,21310133	0,70303320
**IL13**	0,02557373	0,26142036	-0,27605067	0,82584866	-0,27605067	0,58266060
**ST1A1**	0,02557373	0,26142036	-0,91818267	0,52917519	-0,91818267	0,58266060
**CCL25**	0,03015137	0,27739258	-0,12007000	0,92014300	-0,12007000	0,55690516
**IFN-gamma**	0,06372070	0,53293679	-0,18504600	0,87962103	-0,18504600	0,27332430
**IL-24**	0,08325195	0,63826497	0,16562533	1,12165215	0,16562533	0,19499899
**FGF-23**	0,09460449	0,66950871	-0,12879467	0,91459525	-0,12879467	0,17424377
**AXIN1**	0,12054443	0,73933919	-0,92289200	0,52745064	-0,92289200	0,13115627
**CX3CL1**	0,12054443	0,73933919	0,11236000	1,08099511	0,11236000	0,13115627
**FGF-19**	0,15142822	0,81745991	-0,21322933	0,86260421	-0,21322933	0,08753354
**CSF-1**	0,15142822	0,81745991	0,05194133	1,03665894	0,05194133	0,08753354
**VEGFA**	0,16882324	0,81745991	-0,13936267	0,90792015	-0,13936267	0,08753354
**uPA**	0,16882324	0,81745991	0,06787467	1,04817141	0,06787467	0,08753354
**TNFRSF9**	0,18762207	0,85996275	-0,08897733	0,94018897	-0,08897733	0,06552036
**STAMBP**	0,20776367	0,85996275	-0,66320333	0,63147462	-0,66320333	0,06552036
**TNFB**	0,20776367	0,85996275	-0,04696533	0,96797029	-0,04696533	0,06552036
**IL2**	0,22930908	0,85996275	0,13268867	1,09633497	0,13268867	0,06552036
**IL4**	0,22930908	0,85996275	0,19327667	1,14335758	0,19327667	0,06552036
**CST5**	0,25238037	0,85996275	-0,07879067	0,94685101	-0,07879067	0,06552036

**TSLP**	0,25238037	0,85996275	-0,22609933	0,85494331	-0,22609933	0,06552036
**MCP-4**	0,25238037	0,85996275	-0,19279667	0,87490807	-0,19279667	0,06552036
**SIRT2**	0,27685547	0,89364804	-0,58193333	0,66806791	-0,58193333	0,04883350
**CD244**	0,30279541	0,89364804	-0,18343467	0,88060402	-0,18343467	0,04883350
**CXCL9**	0,33026123	0,89364804	-0,01548133	0,98932653	-0,01548133	0,04883350
**TNFSF14**	0,33026123	0,89364804	-0,26108267	0,83446147	-0,26108267	0,04883350
**CCL23**	0,33026123	0,89364804	0,04341067	1,03054726	0,04341067	0,04883350
**LIF**	0,33026123	0,89364804	-0,04342933	0,97034566	-0,04342933	0,04883350
**CASP-8**	0,33026123	0,89364804	-0,41904400	0,74792007	-0,41904400	0,04883350
**IL-18R1**	0,35913086	0,93204510	0,05785600	1,04091770	0,05785600	0,03056307
**IL8**	0,38940430	0,93204510	0,12501267	1,09051731	0,12501267	0,03056307
**CDCP1**	0,38940430	0,93204510	0,06001400	1,04247588	0,06001400	0,03056307
**IL-10RB**	0,38940430	0,93204510	-0,03030600	0,97921258	-0,03030600	0,03056307
**TGF-alpha**	0,42120361	0,93204510	-0,06603867	0,95525733	-0,06603867	0,03056307
**IL-20**	0,42120361	0,93204510	-0,06950200	0,95296689	-0,06950200	0,03056307
**CD40**	0,42120361	0,93204510	-0,21047733	0,86425124	-0,21047733	0,03056307
**IL-10RA**	0,45428467	0,93204510	0,05061267	1,03570466	0,05061267	0,03056307
**CCL19**	0,45428467	0,93204510	0,05142600	1,03628871	0,05142600	0,03056307
**CCL20**	0,48870850	0,93204510	-0,13801533	0,90876846	-0,13801533	0,03056307
**MCP-3**	0,52447510	0,93204510	-0,04253800	0,97094535	-0,04253800	0,03056307
**CD6**	0,52447510	0,93204510	-0,00670867	0,99536070	-0,00670867	0,03056307
**IL5**	0,52447510	0,93204510	-0,05110133	0,96519923	-0,05110133	0,03056307
**IL-20RA**	0,56140137	0,93204510	-0,00010400	0,99992792	-0,00010400	0,03056307
**CCL11**	0,56140137	0,93204510	-0,03443933	0,97641114	-0,03443933	0,03056307
**FGF-21**	0,56140137	0,93204510	-0,14019200	0,90739839	-0,14019200	0,03056307
**PD-L1**	0,56140137	0,93204510	-0,07922533	0,94656578	-0,07922533	0,03056307
**4E-BP1**	0,56140137	0,93204510	-0,25321733	0,83902323	-0,25321733	0,03056307
**CXCL11**	0,59948730	0,93204510	-0,11442733	0,92374891	-0,11442733	0,03056307
**SCF**	0,59948730	0,93204510	0,02845933	1,01992236	0,02845933	0,03056307
**MMP-10**	0,59948730	0,93204510	-0,04450533	0,96962222	-0,04450533	0,03056307
**CCL3**	0,59948730	0,93204510	-0,06328200	0,95708436	-0,06328200	0,03056307
**OPG**	0,63867188	0,93204510	-0,02589667	0,98220994	-0,02589667	0,03056307
**LAP TGF-beta-1**	0,63867188	0,93204510	-0,08079867	0,94553406	-0,08079867	0,03056307
**HGF**	0,63867188	0,93204510	0,08697133	1,06213808	0,08697133	0,03056307
**IL33**	0,63867188	0,93204510	-0,07883400	0,94682257	-0,07883400	0,03056307
**IL-2RB**	0,67877197	0,93204510	0,05613533	1,03967696	0,05613533	0,03056307
**CXCL1**	0,67877197	0,93204510	0,12509467	1,09057929	0,12509467	0,03056307
**SLAMF1**	0,67877197	0,93204510	0,04485400	1,03157878	0,04485400	0,03056307
**Beta-NGF**	0,67877197	0,93204510	-0,01524400	0,98948929	-0,01524400	0,03056307
**IL-12B**	0,67877197	0,93204510	0,01361600	1,00948257	0,01361600	0,03056307
**CD5**	0,67877197	0,93204510	-0,03913867	0,97323583	-0,03913867	0,03056307
**ADA**	0,67877197	0,93204510	-0,07651133	0,94834813	-0,07651133	0,03056307
**IL7**	0,71972656	0,93260343	0,03687000	1,02588570	0,03687000	0,03030299
**TRAIL**	0,71972656	0,93260343	0,04044067	1,02842791	0,04044067	0,03030299

**IL-22 RA1**	0,71972656	0,93260343	0,06991800	1,04965702	0,06991800	0,03030299
**TRANCE**	0,71972656	0,93260343	0,08498667	1,06067794	0,08498667	0,03030299
**CD8A**	0,80395508	0,97654031	0,02312267	1,01615654	0,02312267	0,01030983
**GDNF**	0,80395508	0,97654031	-0,01742333	0,98799570	-0,01742333	0,01030983
**EN-RAGE**	0,80395508	0,97654031	-0,03260933	0,97765047	-0,03260933	0,01030983
**MCP-1**	0,84692383	0,97654031	0,02264067	1,01581710	0,02264067	0,01030983
**CCL4**	0,84692383	0,97654031	-0,01784333	0,98770811	-0,01784333	0,01030983
**CXCL5**	0,84692383	0,97654031	-0,06869200	0,95350209	-0,06869200	0,01030983
**TWEAK**	0,84692383	0,97654031	0,13709800	1,09969085	0,13709800	0,01030983
**FGF-5**	0,89038086	0,97654031	0,10030467	1,07199982	0,10030467	0,01030983
**MCP-2**	0,89038086	0,97654031	-0,00046000	0,99968120	-0,00046000	0,01030983
**NT-3**	0,89038086	0,97654031	0,03241267	1,02272103	0,03241267	0,01030983
**IL-17A**	0,93408203	0,97654031	0,02173267	1,01517797	0,02173267	0,01030983
**IL18**	0,93408203	0,97654031	-0,00437800	0,99697000	-0,00437800	0,01030983
**MMP-1**	0,93408203	0,97654031	-0,03991467	0,97271248	-0,03991467	0,01030983
**LIF-R**	0,93408203	0,97654031	-0,00006333	0,99995610	-0,00006333	0,01030983
**IL-15RA**	0,93408203	0,97654031	-0,01383400	0,99045683	-0,01383400	0,01030983
**CXCL6**	0,93408203	0,97654031	-0,11702333	0,92208820	-0,11702333	0,01030983
**DNER**	0,93408203	0,97654031	0,03256200	1,02282689	0,03256200	0,01030983
**TNF**	0,97796631	0,99969889	0,00716867	1,00498131	0,00716867	0,00013079
**CCL28**	0,97796631	0,99969889	0,29120133	1,22365880	0,29120133	0,00013079
**IL-1 alpha**	1,00000000	1,00000000	-0,01092133	0,99245849	-0,01092133	0,00000000
**ARTN**	1,00000000	1,00000000	-0,00652267	0,99548904	-0,00652267	0,00000000

ESM Table 4: Results of Wilcoxon matched pairs test comparing inflammatory protein levels between baseline (T0) and one day (T1) after adrenaline administration in participants with type 1 diabetes mellitus (T1DM, n=15). Differences were calculated as T1 minus T0. See ESM Table 11 for definitions of OLINK protein abbreviations.

Proteins	P value	FDR	Difference	Fold change	FClog	plog
**HGF**	0,00024414	0,02246094	-1,09266066	0,46889583	-1,09266066	1,64857212
**CCL28**	0,00073242	0,03369141	-0,85164692	0,55415178	-0,85164692	1,47248086
**NRTN**	0,00170898	0,05240885	-0,60722236	0,65645937	-0,60722236	1,28059534
**TWEAK**	0,01049805	0,24145508	-0,35575846	0,78145870	-0,35575846	0,61716366
**CX3CL1**	0,01342773	0,24707031	0,24003038	1,18101753	0,24003038	0,60717944
**ARTN**	0,02148438	0,32942708	-0,19893121	0,87119573	-0,19893121	0,48224070
**TSLP**	0,03271484	0,42996652	-0,24291253	0,84503762	-0,24291253	0,36656536
**CXCL11**	0,03979492	0,45764160	-0,23741192	0,84826567	-0,23741192	0,33947450
**uPA**	0,05737305	0,52783203	0,16956148	1,12471657	0,16956148	0,27750426
**ST1A1**	0,05737305	0,52783203	-0,47271725	0,72060609	-0,47271725	0,27750426
**IL13**	0,06811523	0,56969105	-0,15037527	0,90101606	-0,15037527	0,24436060
**Flt3L**	0,08032227	0,60304214	0,16806379	1,12354958	0,16806379	0,21965234
**SCF**	0,10986328	0,60304214	0,10338824	1,07429353	0,10338824	0,21965234
**MCP-4**	0,10986328	0,60304214	-0,25868016	0,83585224	-0,25868016	0,21965234

**CSF-1**	0,10986328	0,60304214	0,11555093	1,08338869	0,11555093	0,21965234
**IL-10RA**	0,12719727	0,60304214	0,10562143	1,07595775	0,10562143	0,21965234
**CASP-8**	0,12719727	0,60304214	-0,30715159	0,80823594	-0,30715159	0,21965234
**LIF-R**	0,14648438	0,60304214	0,11378538	1,08206366	0,11378538	0,21965234
**CXCL6**	0,14648438	0,60304214	-0,30209538	0,81107353	-0,30209538	0,21965234
**IL-20**	0,14648438	0,60304214	-0,09506341	0,93623110	-0,09506341	0,21965234
**SIRT2**	0,14648438	0,60304214	-0,45554027	0,72923703	-0,45554027	0,21965234
**CXCL9**	0,16772461	0,60304214	-0,05516264	0,96248593	-0,05516264	0,21965234
**CCL4**	0,16772461	0,60304214	-0,12496082	0,91702894	-0,12496082	0,21965234
**VEGFA**	0,19091797	0,60304214	0,15632286	1,11444302	0,15632286	0,21965234
**CD8A**	0,19091797	0,60304214	0,11277797	1,08130834	0,11277797	0,21965234
**AXIN1**	0,19091797	0,60304214	-0,47948264	0,71723478	-0,47948264	0,21965234
**IL-24**	0,19091797	0,60304214	-0,09537176	0,93603102	-0,09537176	0,21965234
**CDCP1**	0,21630859	0,60304214	0,06623104	1,04697794	0,06623104	0,21965234
**OPG**	0,21630859	0,60304214	0,07924929	1,05646816	0,07924929	0,21965234
**IL6**	0,21630859	0,60304214	0,29826368	1,22966359	0,29826368	0,21965234
**IL18**	0,21630859	0,60304214	0,12530665	1,09073955	0,12530665	0,21965234
**STAMBP**	0,21630859	0,60304214	-0,46701308	0,72346089	-0,46701308	0,21965234
**ADA**	0,21630859	0,60304214	-0,07533484	0,94912181	-0,07533484	0,21965234
**MCP-3**	0,24389648	0,60644531	-0,02958973	0,97969887	-0,02958973	0,21720836
**MMP-1**	0,24389648	0,60644531	-0,16278088	0,89330152	-0,16278088	0,21720836
**CCL23**	0,24389648	0,60644531	0,13402231	1,09734891	0,13402231	0,21720836
**CD5**	0,24389648	0,60644531	0,12119527	1,08763560	0,12119527	0,21720836
**IL-22 RA1**	0,27343750	0,61083984	-0,25216231	0,83963703	-0,25216231	0,21407264
**FGF-19**	0,27343750	0,61083984	0,30590093	1,23619037	0,30590093	0,21407264
**MCP-2**	0,27343750	0,61083984	-0,14050407	0,90720213	-0,14050407	0,21407264
**IL-2RB**	0,30541992	0,61083984	-0,12623077	0,91622208	-0,12623077	0,21407264
**FGF-21**	0,30541992	0,61083984	0,21721879	1,16249039	0,21721879	0,21407264
**IL10**	0,30541992	0,61083984	0,17006016	1,12510540	0,17006016	0,21407264
**4E-BP1**	0,30541992	0,61083984	-0,20891115	0,86518997	-0,20891115	0,21407264
**CCL25**	0,30541992	0,61083984	0,08567621	1,06118502	0,08567621	0,21407264
**CCL20**	0,30541992	0,61083984	0,06077813	1,04302818	0,06077813	0,21407264
**IL-10RB**	0,33959961	0,65089925	0,07032374	1,04995226	0,07032374	0,18648623
**TNFRSF9**	0,33959961	0,65089925	0,18894544	1,13993016	0,18894544	0,18648623
**MMP-10**	0,37573242	0,70545679	0,10321319	1,07416319	0,10321319	0,15152958
**CD6**	0,41430664	0,72623698	0,17081077	1,12569093	0,17081077	0,13892164
**TNFSF14**	0,41430664	0,72623698	-0,20392341	0,86818632	-0,20392341	0,13892164
**TRAIL**	0,45483398	0,72623698	0,06324374	1,04481226	0,06324374	0,13892164
**IL-20RA**	0,45483398	0,72623698	-0,01391055	0,99040428	-0,01391055	0,13892164
**SLAMF1**	0,45483398	0,72623698	0,08011500	1,05710230	0,08011500	0,13892164
**FGF-5**	0,45483398	0,72623698	-0,01984560	0,98633826	-0,01984560	0,13892164
**CXCL5**	0,45483398	0,72623698	-0,38607670	0,76520770	-0,38607670	0,13892164
**IL-12B**	0,45483398	0,72623698	-0,05749599	0,96093051	-0,05749599	0,13892164
**IL7**	0,49731445	0,72623698	-0,11014626	0,92649413	-0,11014626	0,13892164

**CXCL1**	0,49731445	0,72623698	-0,13591412	0,91009300	-0,13591412	0,13892164
**CCL19**	0,49731445	0,72623698	0,11203374	1,08075067	0,11203374	0,13892164
**DNER**	0,49731445	0,72623698	0,07606973	1,05414237	0,07606973	0,13892164
**NT-3**	0,49731445	0,72623698	-0,08320863	0,94395590	-0,08320863	0,13892164
**IL5**	0,49731445	0,72623698	-0,10273412	0,93126643	-0,10273412	0,13892164
**TRANCE**	0,54174805	0,76678185	0,08191253	1,05842022	0,08191253	0,11532818
**TNF**	0,54174805	0,76678185	0,09128319	1,06531729	0,09128319	0,11532818
**LAP TGF-beta-1**	0,58789063	0,77265625	-0,08053687	0,94570566	-0,08053687	0,11201368
**IL-15RA**	0,58789063	0,77265625	0,07582676	1,05396485	0,07582676	0,11201368
**IL-18R1**	0,58789063	0,77265625	0,04854275	1,03421974	0,04854275	0,11201368
**CXCL10**	0,58789063	0,77265625	-0,01628978	0,98877229	-0,01628978	0,11201368
**IFN-gamma**	0,58789063	0,77265625	0,03303000	1,02315875	0,03303000	0,11201368
**IL-17C**	0,63549805	0,81202528	0,16403088	1,12041320	0,16403088	0,09043045
**CD40**	0,63549805	0,81202528	-0,04845522	0,96697117	-0,04845522	0,09043045
**CD244**	0,68481445	0,81821987	-0,07302214	0,95064451	-0,07302214	0,08712998
**IL-17A**	0,68481445	0,81821987	0,03643115	1,02557369	0,03643115	0,08712998
**CCL11**	0,68481445	0,81821987	0,02334352	1,01631211	0,02334352	0,08712998
**CCL3**	0,68481445	0,81821987	-0,05559390	0,96219826	-0,05559390	0,08712998
**IL4**	0,68481445	0,81821987	0,07085648	1,05034005	0,07085648	0,08712998
**LIF**	0,73535156	0,86733774	-0,06021000	0,95912450	-0,06021000	0,06181176
**IL8**	0,78686523	0,91634939	0,03933000	1,02763647	0,03933000	0,03793891
**MCP-1**	0,83935547	0,93036992	0,00933176	1,00648925	0,00933176	0,03134434
**OSM**	0,83935547	0,93036992	-0,09107995	0,93881972	-0,09107995	0,03134434
**TGF-alpha**	0,83935547	0,93036992	-0,05617599	0,96181012	-0,05617599	0,03134434
**EN-RAGE**	0,83935547	0,93036992	-0,04503269	0,96926785	-0,04503269	0,03134434
**GDNF**	0,89257813	0,94387572	-0,02663659	0,98170632	-0,02663659	0,02508519
**IL-1 alpha**	0,89257813	0,94387572	0,07854071	1,05594941	0,07854071	0,02508519
**PD-L1**	0,89257813	0,94387572	-0,01014962	0,99298951	-0,01014962	0,02508519
**IL33**	0,89257813	0,94387572	0,03301022	1,02314472	0,03301022	0,02508519
**CST5**	0,94604492	0,98904696	-0,01901456	0,98690659	-0,01901456	0,00478309
**FGF-23**	1,00000000	1,00000000	-0,14294308	0,90566972	-0,14294308	0,00000000
**IL2**	1,00000000	1,00000000	0,00962467	1,00669362	0,00962467	0,00000000
**Beta-NGF**	1,00000000	1,00000000	-0,00763511	0,99472172	-0,00763511	0,00000000
**TNFB**	1,00000000	1,00000000	0,01433527	1,00998599	0,01433527	0,00000000

ESM Table 5: Results of Wilcoxon matched pairs test comparing inflammatory protein levels between baseline (T0) and three days (T3) after adrenaline administration in participants with type 1 diabetes mellitus (T1DM, n=15). Differences were calculated as T3 minus T0. See ESM Table 11 for definitions of OLINK protein abbreviations.

Proteins	P value	FDR	Difference	Fold change	FClog	plog
**uPA**	0,00012207	0,00561523	0,27880400	1,21318873	0,27880400	2,25063211
**HGF**	0,00012207	0,00561523	-0,97109867	0,51011744	-0,97109867	2,25063211
**Flt3L**	0,00018311	0,00561523	0,31222067	1,24161739	0,31222067	2,25063211
**NRTN**	0,00030518	0,00701904	-0,62166467	0,64992058	-0,62166467	2,15372210
**TNFRSF9**	0,00085449	0,01572266	0,24147533	1,18220099	0,24147533	1,80347408
**CCL25**	0,00115967	0,01778158	0,22983400	1,17270001	0,22983400	1,75002976
**CCL28**	0,00262451	0,03449358	-0,66929400	0,62881433	-0,66929400	1,46226170
**SCF**	0,00537109	0,06176758	0,15748400	1,11534033	0,15748400	1,20923943
**OPG**	0,00671387	0,06176758	0,19790800	1,14703388	0,19790800	1,20923943
**CSF-1**	0,00671387	0,06176758	0,19289133	1,14305224	0,19289133	1,20923943
**IL-10RA**	0,01025391	0,08575994	0,14139467	1,10297085	0,14139467	1,06671552
**CDCP1**	0,01507568	0,10668945	0,18736667	1,13868339	0,18736667	0,97187851
**TRAIL**	0,01507568	0,10668945	0,18466533	1,13655329	0,18466533	0,97187851
**VEGFA**	0,01806641	0,11080729	0,28858467	1,22144141	0,28858467	0,95543166
**IL10**	0,01806641	0,11080729	0,34069133	1,26636328	0,34069133	0,95543166
**CST5**	0,02154541	0,11659869	0,20177933	1,15011596	0,20177933	0,93330633
**CX3CL1**	0,02154541	0,11659869	0,24912600	1,18848690	0,24912600	0,93330633
**IL-10RB**	0,03015137	0,14599609	0,12994267	1,09425021	0,12994267	0,83565876
**CD5**	0,03015137	0,14599609	0,15062733	1,11005206	0,15062733	0,83565876
**FGF-21**	0,03533936	0,14778276	0,76435200	1,69860688	0,76435200	0,83037623
**MMP-10**	0,03533936	0,14778276	0,21212067	1,15838969	0,21212067	0,83037623
**CCL20**	0,03533936	0,14778276	0,58342533	1,49840263	0,58342533	0,83037623
**IL18**	0,04125977	0,16503906	0,16733400	1,12298137	0,16733400	0,78241325
**IL-17A**	0,04791260	0,18366496	0,23968400	1,18073401	0,23968400	0,73597370
**LIF-R**	0,05535889	0,18863028	0,17222933	1,12679833	0,17222933	0,72438859
**CCL19**	0,05535889	0,18863028	0,19735133	1,14659138	0,19735133	0,72438859
**IL-12B**	0,05535889	0,18863028	0,15697133	1,11494406	0,15697133	0,72438859
**IL-18R1**	0,06372070	0,20936802	0,15188600	1,11102093	0,15188600	0,67908964
**TGF-alpha**	0,07299805	0,23158001	0,19876133	1,14771253	0,19876133	0,63529893
**CCL11**	0,08325195	0,24707031	0,17313400	1,12750513	0,17313400	0,60717944
**FGF-23**	0,08325195	0,24707031	0,20125933	1,14970150	0,20125933	0,60717944
**SLAMF1**	0,09460449	0,26374586	0,17058867	1,12551764	0,17058867	0,57881435
**IL-15RA**	0,09460449	0,26374586	0,15699333	1,11496107	0,15699333	0,57881435
**TWEAK**	0,10699463	0,28951488	-0,20848733	0,86544417	-0,20848733	0,53832911
**IL6**	0,13537598	0,34596083	0,31270267	1,24203228	0,31270267	0,46097307
**CD6**	0,13537598	0,34596083	0,12985867	1,09418650	0,12985867	0,46097307
**IL-20RA**	0,15142822	0,35721529	0,14411467	1,10505231	0,14411467	0,44706995
**TNF**	0,15142822	0,35721529	0,13127333	1,09525996	0,13127333	0,44706995
**DNER**	0,15142822	0,35721529	0,11429200	1,08244371	0,11429200	0,44706995

**IL-24**	0,16882324	0,38829346	0,24948733	1,18878460	0,24948733	0,41083993
**IL-2RB**	0,18762207	0,41098168	0,17145333	1,12619241	0,17145333	0,38617754
**TNFB**	0,18762207	0,41098168	0,11880000	1,08583132	0,11880000	0,38617754
**IL-1 alpha**	0,22930908	0,45861816	0,17475533	1,12877296	0,17475533	0,33854875
**IL2**	0,22930908	0,45861816	0,19881133	1,14775231	0,19881133	0,33854875
**FGF-5**	0,22930908	0,45861816	0,07921733	1,05644476	0,07921733	0,33854875
**FGF-19**	0,22930908	0,45861816	0,31814400	1,24672563	0,31814400	0,33854875
**GDNF**	0,25238037	0,48372904	0,14637533	1,10678526	0,14637533	0,31539784
**IL4**	0,25238037	0,48372904	0,17612733	1,12984693	0,17612733	0,31539784
**IL-17C**	0,27685547	0,50941406	0,37121800	1,29344436	0,37121800	0,29292907
**EN-RAGE**	0,27685547	0,50941406	0,10223667	1,07343636	0,10223667	0,29292907
**CXCL9**	0,30279541	0,54621917	-0,12083867	0,91965288	-0,12083867	0,26263306
**Beta-NGF**	0,33026123	0,58430833	0,05267933	1,03718938	0,05267933	0,23335792
**CCL23**	0,38940430	0,67594708	0,10175267	1,07307630	0,10175267	0,17008730
**CD8A**	0,42120361	0,69197736	0,07680467	1,05467951	0,07680467	0,15990811
**IL13**	0,42120361	0,69197736	0,10201000	1,07326773	0,10201000	0,15990811
**ADA**	0,42120361	0,69197736	0,05655933	1,03998256	0,05655933	0,15990811
**IL5**	0,45428467	0,73323139	0,04651067	1,03276404	0,04651067	0,13475895
**IL7**	0,48870850	0,74935303	0,11995333	1,08669971	0,11995333	0,12531353
**LAP TGF-beta-1**	0,48870850	0,74935303	0,07106200	1,05048969	0,07106200	0,12531353
**CXCL10**	0,48870850	0,74935303	0,00556533	1,00386505	0,00556533	0,12531353
**MCP-1**	0,52447510	0,77825337	0,09992067	1,07171453	0,09992067	0,10887899
**CXCL1**	0,52447510	0,77825337	0,31896067	1,24743156	0,31896067	0,10887899
**IL-22 RA1**	0,56140137	0,78255948	0,10977067	1,07905669	0,10977067	0,10648264
**CXCL6**	0,56140137	0,78255948	-0,07870400	0,94690789	-0,07870400	0,10648264
**4E-BP1**	0,56140137	0,78255948	-0,23249667	0,85116063	-0,23249667	0,10648264
**STAMBP**	0,56140137	0,78255948	-0,28698067	0,81961559	-0,28698067	0,10648264
**ARTN**	0,59948730	0,79931641	-0,05508533	0,96253751	-0,05508533	0,09728127
**CCL3**	0,59948730	0,79931641	0,10099333	1,07251166	0,10099333	0,09728127
**ST1A1**	0,59948730	0,79931641	-0,15493467	0,89817305	-0,15493467	0,09728127
**OSM**	0,63867188	0,82757482	-0,13501733	0,91065889	-0,13501733	0,08219273
**IL-20**	0,63867188	0,82757482	0,05178400	1,03654590	0,05178400	0,08219273
**IL8**	0,71972656	0,85993304	0,03305533	1,02317671	0,03305533	0,06553537
**MCP-3**	0,71972656	0,85993304	0,04783533	1,03371274	0,04783533	0,06553537
**TNFSF14**	0,71972656	0,85993304	-0,04003667	0,97263023	-0,04003667	0,06553537
**PD-L1**	0,71972656	0,85993304	0,04577467	1,03223730	0,04577467	0,06553537
**LIF**	0,71972656	0,85993304	-0,05200333	0,96459596	-0,05200333	0,06553537
**MCP-2**	0,71972656	0,85993304	0,03164933	1,02218005	0,03164933	0,06553537
**MCP-4**	0,76153564	0,89822153	-0,06480667	0,95607343	-0,06480667	0,04661654
**CD244**	0,80395508	0,90199838	0,03077400	1,02156004	0,03077400	0,04479424
**MMP-1**	0,80395508	0,90199838	0,15129867	1,11056872	0,15129867	0,04479424
**SIRT2**	0,80395508	0,90199838	-0,17653667	0,88482456	-0,17653667	0,04479424
**IFN-gamma**	0,80395508	0,90199838	-0,07403667	0,94997624	-0,07403667	0,04479424
**TRANCE**	0,84692383	0,91667050	0,02792067	1,01954162	0,02792067	0,03778675

**IL33**	0,84692383	0,91667050	0,05025133	1,03544529	0,05025133	0,03778675
**NT-3**	0,84692383	0,91667050	0,12677067	1,09184697	0,12677067	0,03778675
**CD40**	0,89038086	0,95250045	0,01780400	1,01241725	0,01780400	0,02113481
**CXCL11**	0,93408203	0,97654031	-0,00066800	0,99953708	-0,00066800	0,01030983
**CASP-8**	0,93408203	0,97654031	-0,09648133	0,93531139	-0,09648133	0,01030983
**AXIN1**	0,97796631	0,98871319	-0,14640667	0,90349801	-0,14640667	0,00492967
**CCL4**	0,97796631	0,98871319	-0,00154667	0,99892851	-0,00154667	0,00492967
**TSLP**	0,97796631	0,98871319	0,01794867	1,01251878	0,01794867	0,00492967
**CXCL5**	1,00000000	1,00000000	0,23563133	1,17742187	0,23563133	0,00000000

ESM table 6: Results of Wilcoxon matched pairs test comparing inflammatory protein levels between baseline (T0) and seven days (T7) after adrenaline administration in participants with type 1 diabetes mellitus (T1DM, n=15). Differences were calculated as T7 minus T0. See ESM Table 11 for definitions of OLINK protein abbreviations.

Proteins	P value	FDR	Difference	Fold change	FClog	plog
**HGF**	0,00024414	0,01123047	-1,09267357	0,46889163	-1,09267357	1,94960212
**NRTN**	0,00024414	0,01123047	-0,64791214	0,63820325	-0,64791214	1,94960212
**CCL28**	0,00061035	0,01871745	-0,79831071	0,57502209	-0,79831071	1,72775337
**uPA**	0,00122070	0,02246094	0,26777143	1,20394662	0,26777143	1,64857212
**Flt3L**	0,00122070	0,02246094	0,37503786	1,29687358	0,37503786	1,64857212
**VEGFA**	0,00170898	0,02620443	0,29570500	1,22748466	0,29570500	1,58162533
**CX3CL1**	0,00402832	0,05294364	0,23882214	1,18002886	0,23882214	1,27618622
**MMP-10**	0,01342773	0,13726128	0,21984000	1,16460442	0,21984000	0,86245194
**TNFRSF9**	0,01342773	0,13726128	0,19329500	1,14337211	0,19329500	0,86245194
**IL18**	0,01660156	0,14340445	0,22556714	1,16923680	0,22556714	0,84343738
**CDCP1**	0,02026367	0,14340445	0,19703286	1,14633829	0,19703286	0,84343738
**IL-10RB**	0,02026367	0,14340445	0,17349929	1,12779065	0,17349929	0,84343738
**CCL25**	0,02026367	0,14340445	0,20392929	1,15183118	0,20392929	0,84343738
**CCL11**	0,02954102	0,16986084	0,14427286	1,10517349	0,14427286	0,76990673
**LIF-R**	0,02954102	0,16986084	0,15421786	1,11281815	0,15421786	0,76990673
**FGF-21**	0,02954102	0,16986084	0,72419071	1,65197369	0,72419071	0,76990673
**CST5**	0,04187012	0,22659122	0,14442143	1,10528731	0,14442143	0,64475692
**CSF-1**	0,04943848	0,25268555	0,15904214	1,11654558	0,15904214	0,59741960
**MCP-1**	0,05798340	0,26672363	0,20611214	1,15357527	0,20611214	0,57393850
**TWEAK**	0,05798340	0,26672363	-0,28708714	0,81955510	-0,28708714	0,57393850
**IL-18R1**	0,06762695	0,29627046	0,15283429	1,11175145	0,15283429	0,52831165
**TSLP**	0,07849121	0,32823597	-0,33091214	0,79503367	-0,33091214	0,48381382
**DNER**	0,09057617	0,36230469	0,09731071	1,06977746	0,09731071	0,44092605
**CD8A**	0,10400391	0,36801382	0,12333571	1,08925045	0,12333571	0,43413587
**CXCL1**	0,10400391	0,36801382	0,26133929	1,19859087	0,26133929	0,43413587
**CD5**	0,10400391	0,36801382	0,17273786	1,12719558	0,17273786	0,43413587
**OPG**	0,11889648	0,38885498	0,15870357	1,11628358	0,15870357	0,41021233
**Beta-NGF**	0,11889648	0,38885498	0,01991214	1,01389773	0,01991214	0,41021233

**TRAIL**	0,13525391	0,38885498	0,14365786	1,10470247	0,14365786	0,41021233
**SLAMF1**	0,13525391	0,38885498	0,18444500	1,13637973	0,18444500	0,41021233
**IL-15RA**	0,13525391	0,38885498	0,10868357	1,07824391	0,10868357	0,41021233
**TNFB**	0,13525391	0,38885498	0,13044500	1,09463129	0,13044500	0,41021233
**IL-17C**	0,15307617	0,41420611	0,30659357	1,23678401	0,30659357	0,38278350
**NT-3**	0,15307617	0,41420611	-0,22393429	0,85622728	-0,22393429	0,38278350
**CD6**	0,17260742	0,45371094	0,08336143	1,05948373	0,08336143	0,34322075
**IL7**	0,19372559	0,46901984	0,06931929	1,04922151	0,06931929	0,32880879
**SCF**	0,19372559	0,46901984	0,14474214	1,10553304	0,14474214	0,32880879
**ARTN**	0,19372559	0,46901984	-0,17916786	0,88321228	-0,17916786	0,32880879
**IL-12B**	0,21655273	0,51084235	0,13315643	1,09669049	0,13315643	0,29171311
**CXCL9**	0,24121094	0,54125381	-0,20326071	0,86858521	-0,20326071	0,26659903
**IL10**	0,24121094	0,54125381	0,18811214	1,13927193	0,18811214	0,26659903
**FGF-23**	0,26757813	0,56690470	0,11103357	1,08000169	0,11103357	0,24648994
**LIF**	0,26757813	0,56690470	-0,11983143	0,92029518	-0,11983143	0,24648994
**OSM**	0,29577637	0,56690470	-0,31248143	0,80525553	-0,31248143	0,24648994
**FGF-5**	0,29577637	0,56690470	-0,07321143	0,95051979	-0,07321143	0,24648994
**TNF**	0,29577637	0,56690470	0,08380500	1,05980953	0,08380500	0,24648994
**CCL23**	0,29577637	0,56690470	0,09147500	1,06545894	0,09147500	0,24648994
**4E-BP1**	0,29577637	0,56690470	-0,46886643	0,72253209	-0,46886643	0,24648994
**IL-1 alpha**	0,32580566	0,59948242	0,19846714	1,14747852	0,19846714	0,22222355
**CXCL5**	0,32580566	0,59948242	0,16107571	1,11812053	0,16107571	0,22222355
**LAP TGF-beta-1**	0,35754395	0,63257775	0,08801643	1,06290778	0,08801643	0,19888609
**IL5**	0,35754395	0,63257775	0,04739571	1,03339780	0,04739571	0,19888609
**IL-2RB**	0,42626953	0,70029994	0,05375786	1,03796504	0,05375786	0,15471591
**CCL19**	0,42626953	0,70029994	0,06085214	1,04308169	0,06085214	0,15471591
**CXCL10**	0,42626953	0,70029994	-0,17477143	0,88590787	-0,17477143	0,15471591
**ST1A1**	0,42626953	0,70029994	-0,12542429	0,91673440	-0,12542429	0,15471591
**AXIN1**	0,46313477	0,71013997	-0,36712500	0,77532603	-0,36712500	0,14865604
**IL-10RA**	0,46313477	0,71013997	0,01271643	1,00885332	0,01271643	0,14865604
**CXCL6**	0,46313477	0,71013997	-0,14687429	0,90320521	-0,14687429	0,14865604
**EN-RAGE**	0,46313477	0,71013997	0,06342071	1,04494044	0,06342071	0,14865604
**MCP-3**	0,50158691	0,74429026	0,00864500	1,00601025	0,00864500	0,12825766
**IL-20**	0,50158691	0,74429026	-0,05388929	0,96333582	-0,05388929	0,12825766
**CXCL11**	0,54162598	0,75499379	-0,15939929	0,89539782	-0,15939929	0,12205662
**TGF-alpha**	0,54162598	0,75499379	0,07034214	1,04996566	0,07034214	0,12205662
**SIRT2**	0,54162598	0,75499379	-0,32246643	0,79970154	-0,32246643	0,12205662
**STAMBP**	0,54162598	0,75499379	-0,40404357	0,75573714	-0,40404357	0,12205662
**IL13**	0,58300781	0,77734375	-0,10238429	0,93149228	-0,10238429	0,10938689
**FGF-19**	0,58300781	0,77734375	0,21494786	1,16066196	0,21494786	0,10938689
**CCL20**	0,58300781	0,77734375	0,27186929	1,20737119	0,27186929	0,10938689
**CCL3**	0,62573242	0,82239118	-0,00310071	0,99785306	-0,00310071	0,08492155
**IL8**	0,66979980	0,84413126	-0,02300643	0,98417964	-0,02300643	0,07359002
**MMP-1**	0,66979980	0,84413126	0,14425929	1,10516309	0,14425929	0,07359002

**IFN-gamma**	0,66979980	0,84413126	-0,09190643	0,93828205	-0,09190643	0,07359002
**CASP-8**	0,71484375	0,88872466	-0,14881286	0,90199238	-0,14881286	0,05123277
**TNFSF14**	0,76086426	0,92104621	-0,05885429	0,96002622	-0,05885429	0,03571858
**MCP-2**	0,76086426	0,92104621	-0,05208857	0,96453897	-0,05208857	0,03571858
**IL-20RA**	0,80773926	0,95271810	0,08801786	1,06290883	0,08801786	0,02103558
**PD-L1**	0,80773926	0,95271810	-0,00443929	0,99692765	-0,00443929	0,02103558
**IL-24**	0,85522461	0,97757927	0,00965500	1,00671478	0,00965500	0,00984801
**IL33**	0,85522461	0,97757927	0,00534357	1,00371075	0,00534357	0,00984801
**IL-17A**	0,90319824	0,97757927	0,04039571	1,02839586	0,04039571	0,00984801
**CCL4**	0,90319824	0,97757927	-0,05150000	0,96493255	-0,05150000	0,00984801
**MCP-4**	0,90319824	0,97757927	-0,05628429	0,96173792	-0,05628429	0,00984801
**IL-22 RA1**	0,90319824	0,97757927	-0,02402643	0,98348406	-0,02402643	0,00984801
**IL4**	0,90319824	0,97757927	-0,03459500	0,97630580	-0,03459500	0,00984801
**CD244**	0,95153809	0,99478982	-0,08559286	0,94239719	-0,08559286	0,00226867
**IL2**	0,95153809	0,99478982	0,01063214	1,00739686	0,01063214	0,00226867
**CD40**	0,95153809	0,99478982	-0,13718357	0,90929254	-0,13718357	0,00226867
**ADA**	1,00000000	1,00000000	-0,00999000	0,99309938	-0,00999000	0,00000000
**GDNF**	1,00000000	1,00000000	-0,00818714	0,99434118	-0,00818714	0,00000000
**IL6**	1,00000000	1,00000000	-0,01608071	0,98891559	-0,01608071	0,00000000
**TRANCE**	1,00000000	1,00000000	0,06884857	1,04887923	0,06884857	0,00000000

ESM table 7: Results of Wilcoxon matched pairs test comparing inflammatory protein levels between baseline (T0) and 60 minutes (T60) after adrenaline administration in matched controls without diabetes mellitus (CON, n=15). Differences were calculated as T60 minus T0. See ESM Table 11 for definitions of OLINK protein abbreviations.

Proteins	P value	FDR	Difference	Fold change	FClog	plog
**IL6**	0,00006104	0,00187174	1,05536867	2,07824922	1,05536867	2,72775337
**IL10**	0,00006104	0,00187174	1,36077867	2,56823758	1,36077867	2,72775337
**Flt3L**	0,00006104	0,00187174	-0,36997933	0,77379358	-0,36997933	2,72775337
**IL-17C**	0,00018311	0,00421143	-0,34932600	0,78495073	-0,34932600	2,37557085
**CXCL10**	0,00030518	0,00467936	-0,50414400	0,70507860	-0,50414400	2,32981336
**TNFRSF9**	0,00030518	0,00467936	-0,28903267	0,81845065	-0,28903267	2,32981336
**CST5**	0,00061035	0,00802176	-0,24531333	0,84363255	-0,24531333	2,09573015
**CCL25**	0,00115967	0,01333618	-0,24820067	0,84194584	-0,24820067	1,87496850
**IL-12B**	0,00152588	0,01544189	-0,17919400	0,88319628	-0,17919400	1,81129942
**IL-10RB**	0,00201416	0,01544189	-0,13291667	0,91198584	-0,13291667	1,81129942
**MMP-10**	0,00201416	0,01544189	-0,23265400	0,85106782	-0,23265400	1,81129942
**ST1A1**	0,00201416	0,01544189	-0,90028533	0,53578076	-0,90028533	1,81129942
**CSF-1**	0,00335693	0,02375676	-0,16470533	0,89211071	-0,16470533	1,62421277
**CASP-8**	0,00537109	0,03529576	-0,77623867	0,58388710	-0,77623867	1,45227748
**FGF-23**	0,00671387	0,04048880	-0,22393067	0,85622943	-0,22393067	1,39266515
**TRAIL**	0,00836182	0,04048880	-0,16088867	0,89447393	-0,16088867	1,39266515
**OSM**	0,00836182	0,04048880	0,52684933	1,44077927	0,52684933	1,39266515

**FGF-21**	0,00836182	0,04048880	-0,25801667	0,83623674	-0,25801667	1,39266515
**IFN-gamma**	0,00836182	0,04048880	-0,26790200	0,83052644	-0,26790200	1,39266515
**LAP TGF-beta-1**	0,01025391	0,04716797	-0,28376267	0,82144582	-0,28376267	1,32635283
**TNF**	0,01245117	0,04980469	-0,16405067	0,89251563	-0,16405067	1,30272978
**DNER**	0,01245117	0,04980469	-0,15653267	0,89717874	-0,15653267	1,30272978
**ADA**	0,01245117	0,04980469	-0,38846533	0,76394182	-0,38846533	1,30272978
**VEGFA**	0,01507568	0,05779012	-0,20668133	0,86652824	-0,20668133	1,23814640
**OPG**	0,01806641	0,06392728	-0,14755267	0,90278061	-0,14755267	1,19431375
**CCL3**	0,01806641	0,06392728	-0,23150800	0,85174413	-0,23150800	1,19431375
**CDCP1**	0,02154541	0,07079206	-0,16238800	0,89354482	-0,16238800	1,15001544
**SCF**	0,02154541	0,07079206	-0,10563800	0,92939385	-0,10563800	1,15001544
**AXIN1**	0,02557373	0,07589623	-1,18352000	0,44027597	-1,18352000	1,11977978
**LIF-R**	0,02557373	0,07589623	-0,13875600	0,90830202	-0,13875600	1,11977978
**IL-15RA**	0,02557373	0,07589623	-0,15282267	0,89948887	-0,15282267	1,11977978
**CD5**	0,03015137	0,08405836	-0,14759000	0,90275725	-0,14759000	1,07541910
**STAMBP**	0,03015137	0,08405836	-1,19375000	0,43716506	-1,19375000	1,07541910
**CD8A**	0,03533936	0,08787083	-0,14514267	0,90428995	-0,14514267	1,05615527
**CCL11**	0,03533936	0,08787083	-0,15024267	0,90109888	-0,15024267	1,05615527
**SIRT2**	0,03533936	0,08787083	-1,22680933	0,42726133	-1,22680933	1,05615527
**IL5**	0,03533936	0,08787083	-0,21633667	0,86074830	-0,21633667	1,05615527
**IL-18R1**	0,04791260	0,11599892	-0,12713867	0,91564567	-0,12713867	0,93554605
**CXCL9**	0,05535889	0,12732544	-0,11010333	0,92652170	-0,11010333	0,89508482
**CD40**	0,05535889	0,12732544	-0,49145000	0,71130983	-0,49145000	0,89508482
**uPA**	0,06372070	0,13323420	-0,12728600	0,91555217	-0,12728600	0,87538429
**IL18**	0,06372070	0,13323420	-0,13216467	0,91246134	-0,13216467	0,87538429
**PD-L1**	0,06372070	0,13323420	-0,36381933	0,77710458	-0,36381933	0,87538429
**TNFB**	0,06372070	0,13323420	-0,12746933	0,91543583	-0,12746933	0,87538429
**CD6**	0,07299805	0,14599609	-0,17496200	0,88579085	-0,17496200	0,83565876
**CCL19**	0,07299805	0,14599609	-0,13718133	0,90929395	-0,13718133	0,83565876
**MCP-1**	0,08325195	0,15956624	-0,10906667	0,92718770	-0,10906667	0,79705898
**TGF-alpha**	0,08325195	0,15956624	-0,17805000	0,88389690	-0,17805000	0,79705898
**TRANCE**	0,09460449	0,17065908	-0,13894733	0,90818157	-0,13894733	0,76787059
**FGF-19**	0,09460449	0,17065908	-0,28091533	0,82306865	-0,28091533	0,76787059
**CCL20**	0,09460449	0,17065908	-0,13790333	0,90883901	-0,13790333	0,76787059
**MCP-4**	0,10699463	0,18228715	-0,21082800	0,86404119	-0,21082800	0,73924396
**IL33**	0,10699463	0,18228715	0,14060067	1,10236399	0,14060067	0,73924396
**CX3CL1**	0,10699463	0,18228715	-0,07762533	0,94761613	-0,07762533	0,73924396
**IL-10RA**	0,13537598	0,22644709	-0,08185600	0,94484134	-0,08185600	0,64503326
**SLAMF1**	0,15142822	0,24877494	-0,11582133	0,92285677	-0,11582133	0,60419337
**CD244**	0,16882324	0,27248664	-0,28699600	0,81960688	-0,28699600	0,56465479
**IL-20RA**	0,18762207	0,28297099	-0,11465667	0,92360208	-0,11465667	0,54825808
**TSLP**	0,18762207	0,28297099	0,22807867	1,17127405	0,22807867	0,54825808
**CCL4**	0,18762207	0,28297099	-0,10340800	0,93083154	-0,10340800	0,54825808
**4E-BP1**	0,18762207	0,28297099	-0,58049133	0,66873599	-0,58049133	0,54825808

**TNFSF14**	0,20776367	0,30829448	-0,30618600	0,80877707	-0,30618600	0,51103425
**Beta-NGF**	0,22930908	0,33486406	-0,05725800	0,96108904	-0,05725800	0,47513147
**CCL23**	0,25238037	0,36279678	-0,06262600	0,95751965	-0,06262600	0,44033657
**IL8**	0,27685547	0,39185697	-0,13751800	0,90908179	-0,13751800	0,40687242
**CXCL6**	0,30279541	0,42207845	-0,18352533	0,88054868	-0,18352533	0,37460682
**NRTN**	0,33026123	0,45349303	-0,14823800	0,90235186	-0,14823800	0,34342938
**MCP-3**	0,35913086	0,47884115	-0,07380467	0,95012902	-0,07380467	0,31980854
**GDNF**	0,35913086	0,47884115	-0,08299067	0,94409853	-0,08299067	0,31980854
**IL2**	0,38940430	0,50458022	0,09106600	1,06515693	0,09106600	0,29706978
**IL13**	0,38940430	0,50458022	0,08663467	1,06189025	0,08663467	0,29706978
**NT-3**	0,45428467	0,58047485	-0,09623400	0,93547176	-0,09623400	0,23621659
**IL-2RB**	0,48870850	0,60758354	-0,13119533	0,91307462	-0,13119533	0,21639400
**EN-RAGE**	0,48870850	0,60758354	-0,06461200	0,95620245	-0,06461200	0,21639400
**CXCL11**	0,52447510	0,63489091	-0,21000867	0,86453204	-0,21000867	0,19730089
**IL-22 RA1**	0,52447510	0,63489091	0,24581067	1,18575888	0,24581067	0,19730089
**IL-17A**	0,59948730	0,71627055	-0,03206267	0,97802099	-0,03206267	0,14492291
**CXCL5**	0,67877197	0,79046863	-0,07224200	0,95115871	-0,07224200	0,10211536
**HGF**	0,67877197	0,79046863	0,11672400	1,08426996	0,11672400	0,10211536
**FGF-5**	0,71972656	0,81746721	0,01243733	1,00865817	0,01243733	0,08752966
**IL-20**	0,71972656	0,81746721	-0,03939667	0,97306180	-0,03939667	0,08752966
**IL7**	0,76153564	0,84411180	-0,01419600	0,99020834	-0,01419600	0,07360003
**MMP-1**	0,76153564	0,84411180	-0,04640600	0,96834564	-0,04640600	0,07360003
**CCL28**	0,80395508	0,88052223	0,02653933	1,01856591	0,02653933	0,05525968
**ARTN**	0,84692383	0,91667050	-0,05361267	0,96352054	-0,05361267	0,03778675
**MCP-2**	0,89038086	0,94155217	-0,05591067	0,96198702	-0,05591067	0,02615561
**TWEAK**	0,89038086	0,94155217	-0,07057467	0,95225861	-0,07057467	0,02615561
**CXCL1**	0,93408203	0,96556794	0,02704733	1,01892463	0,02704733	0,01521716
**IL-24**	0,93408203	0,96556794	-0,00488933	0,99661671	-0,00488933	0,01521716
**IL-1 alpha**	0,97796631	0,97796631	0,00292800	1,00203160	0,00292800	0,00967611
**IL4**	0,97796631	0,97796631	0,04937133	1,03481390	0,04937133	0,00967611
**LIF**	0,97796631	0,97796631	-0,02049800	0,98589233	-0,02049800	0,00967611

ESM table 8: Results of Wilcoxon matched pairs test comparing inflammatory protein levels between baseline (T0) and one day (T1) after adrenaline administration in matched controls without diabetes mellitus (CON, n=15). Differences were calculated as T1 minus T0. See ESM Table 11 for definitions of OLINK protein abbreviations.

Proteins	P value	FDR	Difference	Fold change	FClog	plog
**HGF**	0,00006104	0,00280762	-0,87704600	0,54448115	-0,87704600	2,55166211
**NRTN**	0,00006104	0,00280762	-0,60624000	0,65690652	-0,60624000	2,55166211
**CCL28**	0,00030518	0,00935872	-0,59883067	0,66028892	-0,59883067	2,02878336
**OSM**	0,00152588	0,03509521	-0,78456333	0,58052764	-0,78456333	1,45475209
**TWEAK**	0,00201416	0,03706055	-0,31096667	0,80610146	-0,31096667	1,43108818
**4E-BP1**	0,00262451	0,04024251	-1,65941200	0,31656815	-1,65941200	1,39531491
**IL6**	0,00671387	0,08823940	0,41027067	1,32893511	0,41027067	1,05433747
**CXCL9**	0,01245117	0,12727865	-0,10712600	0,92843576	-0,10712600	0,89524445
**CX3CL1**	0,01245117	0,12727865	0,16821400	1,12366657	0,16821400	0,89524445
**CASP-8**	0,01507568	0,13869629	-0,67569400	0,62603100	-0,67569400	0,85793516
**SIRT2**	0,02154541	0,18019798	-1,49822133	0,35398955	-1,49822133	0,74425009
**uPA**	0,03533936	0,25009390	0,10157133	1,07294144	0,10157133	0,60189690
**STAMBP**	0,03533936	0,25009390	-1,22523867	0,42772675	-1,22523867	0,60189690
**IL8**	0,04791260	0,29386393	-0,26862800	0,83010860	-0,26862800	0,53185371
**ARTN**	0,04791260	0,29386393	-0,19402133	0,87416569	-0,19402133	0,53185371
**ADA**	0,06372070	0,36639404	-0,36689200	0,77545125	-0,36689200	0,43605160
**TNFSF14**	0,08325195	0,45053998	-0,27008067	0,82927318	-0,27008067	0,34626666
**FGF-21**	0,09460449	0,45808491	0,44336867	1,35977567	0,44336867	0,33905401
**IFN-gamma**	0,09460449	0,45808491	0,29171467	1,22409427	0,29171467	0,33905401
**MCP-1**	0,10699463	0,49217529	-0,14071733	0,90706803	-0,14071733	0,30788019
**IL-20**	0,12054443	0,52809942	-0,09621800	0,93548213	-0,09621800	0,27728431
**MCP-4**	0,15142822	0,63324529	-0,16275533	0,89331734	-0,16275533	0,19842803
**CD5**	0,16882324	0,64715576	0,10183200	1,07313531	0,10183200	0,18899118
**TNFRSF9**	0,16882324	0,64715576	0,07139133	1,05072952	0,07139133	0,18899118
**IL-24**	0,18762207	0,69044922	0,32817200	1,25542166	0,32817200	0,16086826
**CCL11**	0,22930908	0,72058947	-0,05243067	0,96431028	-0,05243067	0,14231209
**IL-22 RA1**	0,22930908	0,72058947	0,29121867	1,22367350	0,29121867	0,14231209
**CCL3**	0,22930908	0,72058947	-0,03583667	0,97546589	-0,03583667	0,14231209
**CXCL6**	0,22930908	0,72058947	-0,18733133	0,87822875	-0,18733133	0,14231209
**LAP TGF-beta-1**	0,25238037	0,72058947	-0,09114200	0,93877934	-0,09114200	0,14231209
**Flt3L**	0,25238037	0,72058947	0,10383800	1,07462850	0,10383800	0,14231209
**CXCL10**	0,25238037	0,72058947	0,00463800	1,00321999	0,00463800	0,14231209
**CCL4**	0,27685547	0,72058947	-0,08878733	0,94031280	-0,08878733	0,14231209
**DNER**	0,27685547	0,72058947	-0,05627333	0,96174522	-0,05627333	0,14231209
**EN-RAGE**	0,27685547	0,72058947	-0,12415333	0,91754236	-0,12415333	0,14231209
**CXCL11**	0,30279541	0,72058947	-0,20357667	0,86839501	-0,20357667	0,14231209
**IL-10RB**	0,30279541	0,72058947	0,04761867	1,03355751	0,04761867	0,14231209
**IL10**	0,33026123	0,72058947	0,13911800	1,10123167	0,13911800	0,14231209
**NT-3**	0,33026123	0,72058947	-0,10253867	0,93139261	-0,10253867	0,14231209

**MMP-10**	0,35913086	0,72058947	-0,05208000	0,96454470	-0,05208000	0,14231209
**IL33**	0,35913086	0,72058947	0,08091067	1,05768547	0,08091067	0,14231209
**FGF-19**	0,35913086	0,72058947	0,16460600	1,12085993	0,16460600	0,14231209
**TNFB**	0,35913086	0,72058947	-0,00529267	0,99633812	-0,00529267	0,14231209
**IL-17A**	0,38940430	0,72058947	0,03828133	1,02688978	0,03828133	0,14231209
**TNF**	0,38940430	0,72058947	0,04585400	1,03229406	0,04585400	0,14231209
**IL5**	0,38940430	0,72058947	-0,10977267	0,92673408	-0,10977267	0,14231209
**OPG**	0,42120361	0,72058947	-0,05762800	0,96084259	-0,05762800	0,14231209
**IL-2RB**	0,42120361	0,72058947	-0,10757533	0,92814664	-0,10757533	0,14231209
**CD6**	0,42120361	0,72058947	0,04266667	1,03001594	0,04266667	0,14231209
**IL4**	0,42120361	0,72058947	-0,12518067	0,91688922	-0,12518067	0,14231209
**LIF**	0,42120361	0,72058947	0,04893800	1,03450312	0,04893800	0,14231209
**MCP-3**	0,45428467	0,72058947	-0,06397333	0,95662584	-0,06397333	0,14231209
**CXCL1**	0,45428467	0,72058947	0,24231867	1,18289225	0,24231867	0,14231209
**TSLP**	0,45428467	0,72058947	-0,11323600	0,92451203	-0,11323600	0,14231209
**IL18**	0,45428467	0,72058947	-0,05792000	0,96064813	-0,05792000	0,14231209
**Beta-NGF**	0,45428467	0,72058947	-0,03431933	0,97649236	-0,03431933	0,14231209
**IL-12B**	0,45428467	0,72058947	-0,06469800	0,95614545	-0,06469800	0,14231209
**CSF-1**	0,45428467	0,72058947	-0,02265800	0,98441736	-0,02265800	0,14231209
**IL-1 alpha**	0,48870850	0,72518035	-0,17878800	0,88344486	-0,17878800	0,13955397
**SLAMF1**	0,48870850	0,72518035	0,04329667	1,03046583	0,04329667	0,13955397
**LIF-R**	0,48870850	0,72518035	0,01680267	1,01171481	0,01680267	0,13955397
**CCL23**	0,48870850	0,72518035	-0,03504267	0,97600290	-0,03504267	0,13955397
**IL-17C**	0,56140137	0,79459886	-0,01626200	0,98879133	-0,01626200	0,09985206
**AXIN1**	0,56140137	0,79459886	-0,21098667	0,86394617	-0,21098667	0,09985206
**CXCL5**	0,56140137	0,79459886	0,24958267	1,18886316	0,24958267	0,09985206
**CST5**	0,59948730	0,82317660	-0,01855867	0,98721850	-0,01855867	0,08450698
**FGF-23**	0,59948730	0,82317660	0,04414467	1,03107170	0,04414467	0,08450698
**CCL19**	0,63867188	0,86408548	0,03843400	1,02699845	0,03843400	0,06344329
**TGF-alpha**	0,67877197	0,86731974	-0,08852000	0,94048706	-0,08852000	0,06182077
**CD40**	0,67877197	0,86731974	0,02248533	1,01570774	0,02248533	0,06182077
**CCL20**	0,67877197	0,86731974	0,16073533	1,11785676	0,16073533	0,06182077
**ST1A1**	0,67877197	0,86731974	-0,13720733	0,90927757	-0,13720733	0,06182077
**CD8A**	0,71972656	0,87124794	-0,04154800	0,97161186	-0,04154800	0,05985823
**GDNF**	0,71972656	0,87124794	-0,04377667	0,97011207	-0,04377667	0,05985823
**IL-10RA**	0,71972656	0,87124794	-0,02779267	0,98091996	-0,02779267	0,05985823
**FGF-5**	0,71972656	0,87124794	-0,00323067	0,99776318	-0,00323067	0,05985823
**CD244**	0,80395508	0,94825471	0,01685733	1,01175314	0,01685733	0,02307499
**MMP-1**	0,80395508	0,94825471	0,09337400	1,06686232	0,09337400	0,02307499
**TRAIL**	0,84692383	0,95020722	-0,01151733	0,99204857	-0,01151733	0,02218167
**IL2**	0,84692383	0,95020722	0,01551733	1,01081385	0,01551733	0,02218167
**IL-15RA**	0,84692383	0,95020722	-0,02547267	0,98249865	-0,02547267	0,02218167
**IL13**	0,84692383	0,95020722	-0,03864600	0,97356823	-0,03864600	0,02218167
**IL-18R1**	0,89038086	0,97517904	-0,00568400	0,99606790	-0,00568400	0,01091564

**TRANCE**	0,89038086	0,97517904	0,01967667	1,01373226	0,01967667	0,01091564
**SCF**	0,93408203	0,97654031	-0,02124067	0,98538494	-0,02124067	0,01030983
**PD-L1**	0,93408203	0,97654031	0,02634533	1,01842895	0,02634533	0,01030983
**MCP-2**	0,93408203	0,97654031	0,01733533	1,01208842	0,01733533	0,01030983
**CCL25**	0,93408203	0,97654031	-0,00141600	0,99901899	-0,00141600	0,01030983
**VEGFA**	0,97796631	0,98871319	0,00257800	1,00178853	0,00257800	0,00492967
**IL-20RA**	0,97796631	0,98871319	0,00080400	1,00055745	0,00080400	0,00492967
**IL7**	0,97796631	0,98871319	0,01442333	1,01004763	0,01442333	0,00492967
**CDCP1**	1,00000000	1,00000000	-0,00559400	0,99613004	-0,00559400	0,00000000

ESM table 9: Results of Wilcoxon matched pairs test comparing inflammatory protein levels between baseline (T0) and three days (T3) after adrenaline administration in matched controls without diabetes mellitus (CON, n=15). Differences were calculated as T3 minus T0. See ESM Table 11 for definitions of OLINK protein abbreviations.

Proteins	P value	FDR	Difference	Fold change	FClog	plog
**HGF**	0,00024414	0,01123047	-0,90106714	0,53549049	-0,90106714	1,94960212
**IL33**	0,00024414	0,01123047	0,30672357	1,23689546	0,30672357	1,94960212
**NRTN**	0,00036621	0,01123047	-0,61750143	0,65179879	-0,61750143	1,94960212
**TWEAK**	0,00524902	0,12072754	-0,39349286	0,76128425	-0,39349286	0,91819365
**CCL28**	0,01074219	0,19765625	-0,64768857	0,63830216	-0,64768857	0,70408945
**IL-20RA**	0,02453613	0,37622070	-0,19648286	0,87267547	-0,19648286	0,42455731
**IL-22 RA1**	0,04187012	0,42800564	0,37980143	1,30116275	0,37980143	0,36855051
**4E-BP1**	0,04187012	0,42800564	-1,38212357	0,38365366	-1,38212357	0,36855051
**ADA**	0,04187012	0,42800564	-0,43885429	0,73772024	-0,43885429	0,36855051
**CXCL9**	0,05798340	0,48141276	-0,25949429	0,83538070	-0,25949429	0,31748240
**MMP-10**	0,06762695	0,48141276	-0,14608786	0,90369769	-0,14608786	0,31748240
**STAMBP**	0,06762695	0,48141276	-1,26671000	0,41560646	-1,26671000	0,31748240
**FGF-23**	0,07849121	0,48141276	0,28563500	1,21894666	0,28563500	0,31748240
**IFN-gamma**	0,07849121	0,48141276	0,32326429	1,25115826	0,32326429	0,31748240
**CASP-8**	0,07849121	0,48141276	-0,66203857	0,63198465	-0,66203857	0,31748240
**SIRT2**	0,09057617	0,52081299	-1,35780929	0,39017432	-1,35780929	0,28331819
**OSM**	0,11889648	0,56332031	-0,53836000	0,68855319	-0,53836000	0,24924459
**Beta-NGF**	0,11889648	0,56332031	-0,08300214	0,94409102	-0,08300214	0,24924459
**uPA**	0,13525391	0,56332031	0,10227929	1,07346807	0,10227929	0,24924459
**IL-1 alpha**	0,13525391	0,56332031	-0,36129357	0,77846627	-0,36129357	0,24924459
**CCL23**	0,13525391	0,56332031	-0,19910286	0,87109209	-0,19910286	0,24924459
**DNER**	0,13525391	0,56332031	-0,10154929	0,93203156	-0,10154929	0,24924459
**IL8**	0,15307617	0,56332031	-0,30032143	0,81207145	-0,30032143	0,24924459
**MCP-4**	0,15307617	0,56332031	-0,24934786	0,84127661	-0,24934786	0,24924459
**CX3CL1**	0,15307617	0,56332031	0,09968571	1,07154001	0,09968571	0,24924459
**IL-10RA**	0,17260742	0,61076472	-0,02850714	0,98043430	-0,02850714	0,21412605
**CXCL6**	0,19372559	0,66010200	-0,32336857	0,79920163	-0,32336857	0,18038895
**CD8A**	0,21655273	0,68699488	-0,11542429	0,92311079	-0,11542429	0,16304650

**CXCL11**	0,21655273	0,68699488	-0,50187357	0,70618909	-0,50187357	0,16304650
**IL-20**	0,24121094	0,72403493	-0,11914786	0,92073133	-0,11914786	0,14024048
**TGF-alpha**	0,26757813	0,72403493	-0,15266786	0,89958539	-0,15266786	0,14024048
**IL-15RA**	0,26757813	0,72403493	-0,07461786	0,94959362	-0,07461786	0,14024048
**ARTN**	0,26757813	0,72403493	-0,15851143	0,89594903	-0,15851143	0,14024048
**CD5**	0,26757813	0,72403493	0,07085429	1,05033845	0,07085429	0,14024048
**MCP-1**	0,35754395	0,86563271	-0,13408929	0,91124488	-0,13408929	0,06266634
**TRAIL**	0,35754395	0,86563271	-0,07322143	0,95051320	-0,07322143	0,06266634
**TSLP**	0,35754395	0,86563271	-0,21387929	0,86221569	-0,21387929	0,06266634
**TNFSF14**	0,35754395	0,86563271	-0,16461357	0,89216746	-0,16461357	0,06266634
**IL7**	0,42626953	0,92850617	-0,11351643	0,92433234	-0,11351643	0,03221520
**CST5**	0,42626953	0,92850617	-0,06306071	0,95723117	-0,06306071	0,03221520
**IL5**	0,42626953	0,92850617	-0,07200071	0,95131781	-0,07200071	0,03221520
**CDCP1**	0,46313477	0,92850617	-0,08159786	0,94501042	-0,08159786	0,03221520
**IL18**	0,50158691	0,92850617	-0,03981786	0,97277775	-0,03981786	0,03221520
**LIF-R**	0,50158691	0,92850617	-0,06030786	0,95905944	-0,06030786	0,03221520
**IL4**	0,50158691	0,92850617	-0,09241571	0,93795089	-0,09241571	0,03221520
**IL6**	0,50969321	0,92850617	0,02926643	1,02049310	0,02926643	0,03221520
**IL2**	0,54162598	0,92850617	-0,08599714	0,94213314	-0,08599714	0,03221520
**SCF**	0,54162598	0,92850617	0,02587071	1,01809396	0,02587071	0,03221520
**IL-12B**	0,54162598	0,92850617	-0,06299000	0,95727809	-0,06299000	0,03221520
**FGF-19**	0,54162598	0,92850617	0,19098357	1,14154171	0,19098357	0,03221520
**LIF**	0,54162598	0,92850617	-0,05625643	0,96175649	-0,05625643	0,03221520
**LAP TGF-beta-1**	0,58300781	0,92850617	-0,11683143	0,92221087	-0,11683143	0,03221520
**IL-2RB**	0,58300781	0,92850617	-0,04715500	0,96784304	-0,04715500	0,03221520
**TRANCE**	0,58300781	0,92850617	-0,12565929	0,91658508	-0,12565929	0,03221520
**IL-24**	0,58300781	0,92850617	0,11374357	1,08203230	0,11374357	0,03221520
**NT-3**	0,58300781	0,92850617	-0,14467929	0,90458045	-0,14467929	0,03221520
**CSF-1**	0,58300781	0,92850617	-0,04514643	0,96919144	-0,04514643	0,03221520
**MCP-3**	0,62573242	0,92850617	-0,12816643	0,91499361	-0,12816643	0,03221520
**GDNF**	0,62573242	0,92850617	-0,07986857	0,94614384	-0,07986857	0,03221520
**FGF-21**	0,62573242	0,92850617	0,07232000	1,05140609	0,07232000	0,03221520
**CXCL5**	0,62573242	0,92850617	-0,19178857	0,87551963	-0,19178857	0,03221520
**Flt3L**	0,62573242	0,92850617	0,02091357	1,01460176	0,02091357	0,03221520
**TNF**	0,66979980	0,96283722	-0,06382357	0,95672515	-0,06382357	0,01644713
**CCL20**	0,66979980	0,96283722	0,07878143	1,05612561	0,07878143	0,01644713
**OPG**	0,71484375	0,98350830	-0,06250571	0,95759949	-0,06250571	0,00722197
**MCP-2**	0,71484375	0,98350830	-0,06067571	0,95881493	-0,06067571	0,00722197
**CCL4**	0,76086426	0,98350830	-0,02756714	0,98107331	-0,02756714	0,00722197
**IL13**	0,76086426	0,98350830	0,05670286	1,04008602	0,05670286	0,00722197
**CCL3**	0,76086426	0,98350830	-0,00423357	0,99706981	-0,00423357	0,00722197
**CXCL10**	0,76086426	0,98350830	-0,06900143	0,95329760	-0,06900143	0,00722197
**TNFB**	0,76086426	0,98350830	-0,05838143	0,96034093	-0,05838143	0,00722197
**FGF-5**	0,80773926	0,98350830	-0,03487500	0,97611633	-0,03487500	0,00722197

**AXIN1**	0,85522461	0,98350830	-0,25525429	0,83783945	-0,25525429	0,00722197
**CXCL1**	0,85522461	0,98350830	-0,13046786	0,91353515	-0,13046786	0,00722197
**CD6**	0,85522461	0,98350830	-0,02108571	0,98549078	-0,02108571	0,00722197
**MMP-1**	0,85522461	0,98350830	-0,06821071	0,95382023	-0,06821071	0,00722197
**IL-18R1**	0,85522461	0,98350830	-0,03959000	0,97293141	-0,03959000	0,00722197
**IL10**	0,85522461	0,98350830	-0,00875143	0,99395233	-0,00875143	0,00722197
**EN-RAGE**	0,85522461	0,98350830	0,01728071	1,01205010	0,01728071	0,00722197
**ST1A1**	0,85522461	0,98350830	-0,19296286	0,87480729	-0,19296286	0,00722197
**IL-17A**	0,90319824	1,00000000	0,02632000	1,01841107	0,02632000	0,00000000
**IL-10RB**	0,90319824	1,00000000	0,01063286	1,00739736	0,01063286	0,00000000
**VEGFA**	0,95153809	1,00000000	-0,01605429	0,98893370	-0,01605429	0,00000000
**SLAMF1**	0,95153809	1,00000000	-0,02503714	0,98279530	-0,02503714	0,00000000
**CCL19**	0,95153809	1,00000000	-0,02873786	0,98027752	-0,02873786	0,00000000
**CCL25**	0,95153809	1,00000000	0,00117071	1,00081181	0,00117071	0,00000000
**TNFRSF9**	0,95153809	1,00000000	-0,01327643	0,99083969	-0,01327643	0,00000000
**CCL11**	1,00000000	1,00000000	-0,03130643	0,97853379	-0,03130643	0,00000000
**PD-L1**	1,00000000	1,00000000	-0,21929929	0,85898254	-0,21929929	0,00000000
**CD40**	1,00000000	1,00000000	-0,16124643	0,89425214	-0,16124643	0,00000000
**CD244**	1,00000000	1,00000000	-0,16356571	0,89281569	-0,16356571	0,00000000
**IL-17C**	1,00000000	1,00000000	0,01260286	1,00877390	0,01260286	0,00000000

ESM table 10: Results of Wilcoxon matched pairs test comparing inflammatory protein levels between baseline (T0) and seven days (T7) after adrenaline administration in matched controls without diabetes mellitus (CON, n = 15). Differences were calculated as T7 minus T0. See ESM Table 11 for definitions of OLINK protein abbreviations.

Proteins	P value	FDR	Difference	Fold change	FClog	plog
**HGF**	0,00012207	0,01123047	-0,86379143	0,54950655	-0,86379143	1,94960212
**NRTN**	0,00024414	0,01123047	-0,55316357	0,68152403	-0,55316357	1,94960212
**FGF-21**	0,00305176	0,07019043	0,70697357	1,63237619	0,70697357	1,15372210
**CCL28**	0,00305176	0,07019043	-0,56820929	0,67445342	-0,56820929	1,15372210
**OSM**	0,00524902	0,08048503	-0,83926071	0,55892991	-0,83926071	1,09428491
**IL33**	0,00524902	0,08048503	0,23583429	1,17758752	0,23583429	1,09428491
**4E-BP1**	0,00671387	0,08823940	-1,63539929	0,32188131	-1,63539929	1,05433747
**uPA**	0,02954102	0,32100423	0,20361857	1,15158314	0,20361857	0,49348924
**Flt3L**	0,03527832	0,32100423	0,16665857	1,12245575	0,16665857	0,49348924
**CX3CL1**	0,03527832	0,32100423	0,20550500	1,15308990	0,20550500	0,49348924
**SIRT2**	0,04187012	0,32100423	-1,41754286	0,37434935	-1,41754286	0,49348924
**CASP-8**	0,04187012	0,32100423	-0,68638929	0,62140714	-0,68638929	0,49348924
**TWEAK**	0,04943848	0,34987230	-0,23844500	0,84765846	-0,23844500	0,45609045
**CXCL9**	0,05798340	0,35563151	-0,31143786	0,80583822	-0,31143786	0,44899977
**ADA**	0,05798340	0,35563151	-0,38592214	0,76528968	-0,38592214	0,44899977
**IL-20RA**	0,07849121	0,45132446	-0,10456071	0,93008810	-0,10456071	0,34551113

**CCL11**	0,09057617	0,46294488	0,14940357	1,10911086	0,14940357	0,33447072
**STAMBP**	0,09057617	0,46294488	-1,25766429	0,41822051	-1,25766429	0,33447072
**FGF-23**	0,10400391	0,50359786	0,15225714	1,11130679	0,15225714	0,29791612
**CD5**	0,11889648	0,54692383	0,12461643	1,09021784	0,12461643	0,26207316
**IFN-gamma**	0,15307617	0,64013672	0,28632429	1,21952919	0,28632429	0,19372726
**TNFRSF9**	0,15307617	0,64013672	0,09397286	1,06730526	0,09397286	0,19372726
**IL6**	0,17260742	0,69042969	0,14222143	1,10360311	0,14222143	0,16088054
**CCL25**	0,19372559	0,71291016	0,08590786	1,06135542	0,08590786	0,14696520
**CCL20**	0,19372559	0,71291016	0,63122000	1,54887423	0,63122000	0,14696520
**TNFSF14**	0,24121094	0,85351563	-0,32126286	0,80036897	-0,32126286	0,06878852
**IL8**	0,29577637	0,87929047	-0,20877714	0,86527034	-0,20877714	0,05586763
**MCP-3**	0,32580566	0,87929047	0,12739286	1,09231795	0,12739286	0,05586763
**CDCP1**	0,32580566	0,87929047	0,07164000	1,05091064	0,07164000	0,05586763
**SLAMF1**	0,32580566	0,87929047	0,09806500	1,07033692	0,09806500	0,05586763
**MMP-10**	0,32580566	0,87929047	-0,12068286	0,91975221	-0,12068286	0,05586763
**CXCL6**	0,32580566	0,87929047	-0,22098286	0,85798073	-0,22098286	0,05586763
**CST5**	0,35754395	0,87929047	0,02923786	1,02047289	0,02923786	0,05586763
**IL-22 RA1**	0,35754395	0,87929047	0,32227500	1,25030061	0,32227500	0,05586763
**NT-3**	0,35754395	0,87929047	-0,20878786	0,86526392	-0,20878786	0,05586763
**CD6**	0,39099121	0,87929047	0,10122214	1,07268177	0,10122214	0,05586763
**LIF**	0,39099121	0,87929047	0,04048857	1,02846206	0,04048857	0,05586763
**MCP-4**	0,42626953	0,87929047	-0,13807500	0,90873087	-0,13807500	0,05586763
**TRANCE**	0,42626953	0,87929047	0,03158286	1,02213295	0,03158286	0,05586763
**IL-12B**	0,42626953	0,87929047	0,05934000	1,04198897	0,05934000	0,05586763
**IL-20**	0,42626953	0,87929047	-0,06847857	0,95364316	-0,06847857	0,05586763
**EN-RAGE**	0,42626953	0,87929047	0,12175857	1,08806034	0,12175857	0,05586763
**OPG**	0,46313477	0,87929047	0,04476214	1,03151310	0,04476214	0,05586763
**CXCL1**	0,46313477	0,87929047	0,29672500	1,22835282	0,29672500	0,05586763
**CCL23**	0,46313477	0,87929047	-0,09106286	0,93883084	-0,09106286	0,05586763
**CD40**	0,46313477	0,87929047	0,00043786	1,00030355	0,00043786	0,05586763
**IL5**	0,46313477	0,87929047	0,01845143	1,01287169	0,01845143	0,05586763
**GDNF**	0,50158691	0,87929047	-0,02896643	0,98012222	-0,02896643	0,05586763
**IL-10RB**	0,50158691	0,87929047	0,04266286	1,03001322	0,04266286	0,05586763
**IL4**	0,50158691	0,87929047	0,13465714	1,09783188	0,13465714	0,05586763
**CD8A**	0,54162598	0,87929047	0,05689571	1,04022507	0,05689571	0,05586763
**CD244**	0,54162598	0,87929047	-0,02326357	0,98400423	-0,02326357	0,05586763
**CXCL11**	0,54162598	0,87929047	-0,23358000	0,85052173	-0,23358000	0,05586763
**CXCL5**	0,54162598	0,87929047	0,25430286	1,19275924	0,25430286	0,05586763
**CXCL10**	0,54162598	0,87929047	0,05935357	1,04199877	0,05935357	0,05586763
**IL-1 alpha**	0,58300781	0,87929047	-0,22317357	0,85667888	-0,22317357	0,05586763
**IL-10RA**	0,58300781	0,87929047	0,05951643	1,04211640	0,05951643	0,05586763
**CCL19**	0,58300781	0,87929047	0,04636429	1,03265925	0,04636429	0,05586763
**IL-18R1**	0,58300781	0,87929047	0,05058143	1,03568224	0,05058143	0,05586763
**Beta-NGF**	0,58300781	0,87929047	-0,03777786	0,97415426	-0,03777786	0,05586763

**IL10**	0,58300781	0,87929047	0,01687571	1,01176604	0,01687571	0,05586763
**IL13**	0,62573242	0,89949036	0,08305714	1,05926029	0,08305714	0,04600349
**ARTN**	0,62573242	0,89949036	-0,06383786	0,95671568	-0,06383786	0,04600349
**DNER**	0,62573242	0,89949036	-0,03006857	0,97937375	-0,03006857	0,04600349
**LAP TGF-beta-1**	0,66979980	0,93366033	-0,07040286	0,95237202	-0,07040286	0,02981109
**IL-17C**	0,66979980	0,93366033	0,12604143	1,09129521	0,12604143	0,02981109
**VEGFA**	0,71484375	0,96714154	0,04617000	1,03252020	0,04617000	0,01450996
**MCP-1**	0,71484375	0,96714154	0,00926643	1,00644367	0,00926643	0,01450996
**IL18**	0,76086426	0,97221544	0,02219071	1,01550033	0,02219071	0,01223749
**IL-15RA**	0,76086426	0,97221544	0,00368929	1,00256049	0,00368929	0,01223749
**PD-L1**	0,76086426	0,97221544	-0,03623429	0,97519708	-0,03623429	0,01223749
**TNFB**	0,76086426	0,97221544	0,03388000	1,02376174	0,03388000	0,01223749
**CCL4**	0,80773926	0,99478982	-0,05364214	0,96350086	-0,05364214	0,00226867
**TNF**	0,80773926	0,99478982	0,02618143	1,01831325	0,02618143	0,00226867
**IL7**	0,85522461	0,99478982	-0,00644214	0,99554460	-0,00644214	0,00226867
**IL2**	0,85522461	0,99478982	0,02568429	1,01796241	0,02568429	0,00226867
**IL-17A**	0,90319824	0,99478982	-0,10677643	0,92866075	-0,10677643	0,00226867
**TRAIL**	0,90319824	0,99478982	-0,00867929	0,99400204	-0,00867929	0,00226867
**TSLP**	0,90319824	0,99478982	0,13346571	1,09692563	0,13346571	0,00226867
**SCF**	0,90319824	0,99478982	-0,01271500	0,99122536	-0,01271500	0,00226867
**IL-24**	0,90319824	0,99478982	0,13171214	1,09559314	0,13171214	0,00226867
**CSF-1**	0,90319824	0,99478982	0,01264143	1,00880087	0,01264143	0,00226867
**AXIN1**	0,95153809	0,99478982	-0,17997214	0,88272004	-0,17997214	0,00226867
**TGF-alpha**	0,95153809	0,99478982	-0,04561714	0,96887527	-0,04561714	0,00226867
**MMP-1**	0,95153809	0,99478982	0,03169000	1,02220886	0,03169000	0,00226867
**LIF-R**	0,95153809	0,99478982	0,02286571	1,01597557	0,02286571	0,00226867
**CCL3**	0,95153809	0,99478982	-0,00986857	0,99318297	-0,00986857	0,00226867
**MCP-2**	0,95153809	0,99478982	-0,02305929	0,98414358	-0,02305929	0,00226867
**IL-2RB**	1,00000000	1,00000000	0,04317429	1,03037842	0,04317429	0,00000000
**FGF-5**	1,00000000	1,00000000	-0,02995643	0,97944988	-0,02995643	0,00000000
**ST1A1**	1,00000000	1,00000000	-0,02590643	0,98220330	-0,02590643	0,00000000
**FGF-19**	1,00000000	1,00000000	-0,01613071	0,98888132	-0,01613071	0,00000000

ESM table 11: Abbreviations OLINK proteins [1]

UniProt ID	Gene	Protein name
**P30203**	CD6	T-cell differentiation antigen CD6
**P21583**	KITLG	Kit ligand
**Q14116**	IL18	Interleukin-18
**Q13291**	SLAMF1	Signaling lymphocytic activation molecule
**P01135**	TGFA	Protransforming growth factor alpha
**Q99616**	CCL13	C-C motif chemokine 13
**P51671**	CCL11	Eotaxin
**O43557**	TNFSF14	Tumor necrosis factor ligand superfamily member 14
**Q9GZV9**	FGF23	Fibroblast growth factor 23
**Q13651**	IL10RA	Interleukin-10 receptor subunit alpha
**P13236**	CCL4	C-C motif chemokine 4
**P12034**	FGF5	Fibroblast growth factor 5
**P42702**	LIFR	Leukemia inhibitory factor receptor
**Q9NSA1**	FGF21	Fibroblast growth factor 21
**Q99731**	CCL19	C-C motif chemokine 19
**Q13261**	IL15RA	Interleukin-15 receptor subunit alpha
**Q08334**	IL10RB	Interleukin-10 receptor subunit beta
**Q8N6P7**	IL22RA1	Interleukin-22 receptor subunit alpha-1
**Q13478**	IL18R1	Interleukin-18 receptor 1
**Q9NZQ7**	CD274	Programmed cell death 1 ligand 1
**P01138**	NGF	Beta-nerve growth factor
**P42830**	CXCL5	C-X-C motif chemokine 5
**P03956**	MMP1	Interstitial collagenase
**O14788**	TNFSF11	Tumor necrosis factor ligand superfamily member 11
**Q969D9**	TSLP	Thymic stromal lymphopoietin
**P60568**	IL2	Interleukin-2
**P15692**	VEGFA	Vascular endothelial growth factor A
**P80098**	CCL7	C-C motif chemokine 7
**P39905**	GDNF	Glial cell line-derived neurotrophic factor
**Q9H5V8**	CDCP1	CUB domain-containing protein 1
**Q9BZW8**	CD244	Natural killer cell receptor 2B4

**P13232**	IL7	Interleukin-7
**O00300**	TNFRSF11B	Tumor necrosis factor receptor superfamily member 11B
**P01137**	TGFB1	Transforming growth factor beta-1 proprotein
**P00749**	PLAU	Urokinase-type plasminogen activator
**P05231**	IL6	Interleukin-6
**P09341**	CXCL1	Growth-regulated alpha protein
**Q9P0M4**	IL17C	Interleukin-17C
**Q16552**	IL17A	Interleukin-17A
**O14625**	CXCL11	C-X-C motif chemokine 11
**O15169**	AXIN1	Axin-1
**P50591**	TNFSF10	Tumor necrosis factor ligand superfamily member 10
**Q9UHF4**	IL20RA	Interleukin-20 receptor subunit alpha
**Q07325**	CXCL9	C-X-C motif chemokine 9
**P28325**	CST5	Cystatin-D
**P14784**	IL2RB	Interleukin-2 receptor subunit beta
**P01583**	IL1A	Interleukin-1 alpha
**P13725**	OSM	Oncostatin-M
**P13500**	CCL2	C-C motif chemokine 2
**P10145**	CXCL8	Interleukin-8
**Q8NFT8**	DNER	Delta and Notch-like epidermal growth factor-related receptor
**Q9NRJ3**	CCL28	C-C motif chemokine 28
**Q8IXJ6**	SIRT2	NAD-dependent protein deacetylase sirtuin-2
**Q9NYY1**	IL20	Interleukin-20
**Q13541**	EIF4EBP1	Eukaryotic translation initiation factor 4E-binding protein 1
**P02778**	CXCL10	C-X-C motif chemokine 10
**P80162**	CXCL6	C-X-C motif chemokine 6
**P49771**	FLT3LG	Fms-related tyrosine kinase 3 ligand
**P80511**	S100A12	Protein S100-A12
**P10147**	CCL3	C-C motif chemokine 3
**P55773**	CCL23	C-C motif chemokine 23
**P01375**	TNF	Tumor necrosis factor
**P22301**	IL10	Interleukin-10
**P09238**	MMP10	Stromelysin-2

**Q5T4W7**	ARTN	Artemin
**P35225**	IL13	Interleukin-13
**Q13007**	IL24	Interleukin-24
**P29460**	IL12B	Interleukin-12 subunit beta
**P06127**	CD5	T-cell surface glycoprotein CD5
**P14210**	HGF	Hepatocyte growth factor
**P25942**	CD40	Tumor necrosis factor receptor superfamily member 5
**P01579**	IFNG	Interferon gamma
**P09603**	CSF1	Macrophage colony-stimulating factor 1
**P01374**	LTA	Lymphotoxin-alpha
**P00813**	ADA	Adenosine deaminase
**P05113**	IL5	Interleukin-5
**O95630**	STAMBP	STAM-binding protein
**P50225**	SULT1A1	Sulfotransferase 1A1
**P78556**	CCL20	C-C motif chemokine 20
**O43508**	TNFSF12	Tumor necrosis factor ligand superfamily member 12
**O95760**	IL33	Interleukin-33
**P20783**	NTF3	Neurotrophin-3
**P78423**	CX3CL1	Fractalkine
**O15444**	CCL25	C-C motif chemokine 25
**Q14790**	CASP8	Caspase-8
**P80075**	CCL8	C-C motif chemokine 8
**Q99748**	NRTN	Neurturin
**P15018**	LIF	Leukemia inhibitory factor
**P05112**	IL4	Interleukin-4
**O95750**	FGF19	Fibroblast growth factor 19
**Q07011**	TNFRSF9	Tumor necrosis factor receptor superfamily member 9
**P01732**	CD8A	T-cell surface glycoprotein CD8 alpha chain

ESM Fig. 1: Adrenaline levels after adrenaline administration versus hypoglycaemia

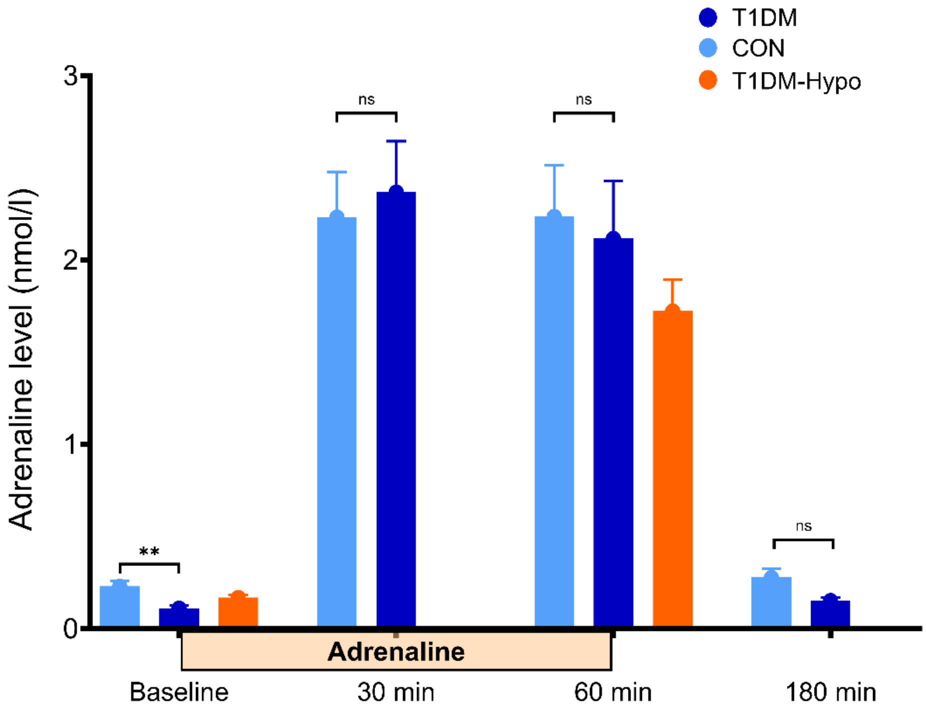

ESM Figure 1*: Adrenaline levels in nmol/L after adrenaline administration for participants with type 1 diabetes mellitus (T1DM; dark blue bar, n=15), matched control participants without diabetes (CON; light blue bar, n=15) and participants with type 1 diabetes mellitus after a hypoglycaemic clamp (T1DM-Hypo: Orange bar, n=47). Timepoints 30 min and 180 min are missing for T1DM-Hypo as adrenaline was not measured at these timepoints. The data visualized by the orange bar (n=47) is data derived from a different cohort, (Verhulst and van Heck et al [8]) with recruitment and execution of experiments done in the period between August 2019 and March 2021. Data are presented as mean ± SEM; *p < 0.05, **p <0.01.*

ESM Fig. 2: Noradrenaline levels after adrenaline administration versus hypoglycaemia

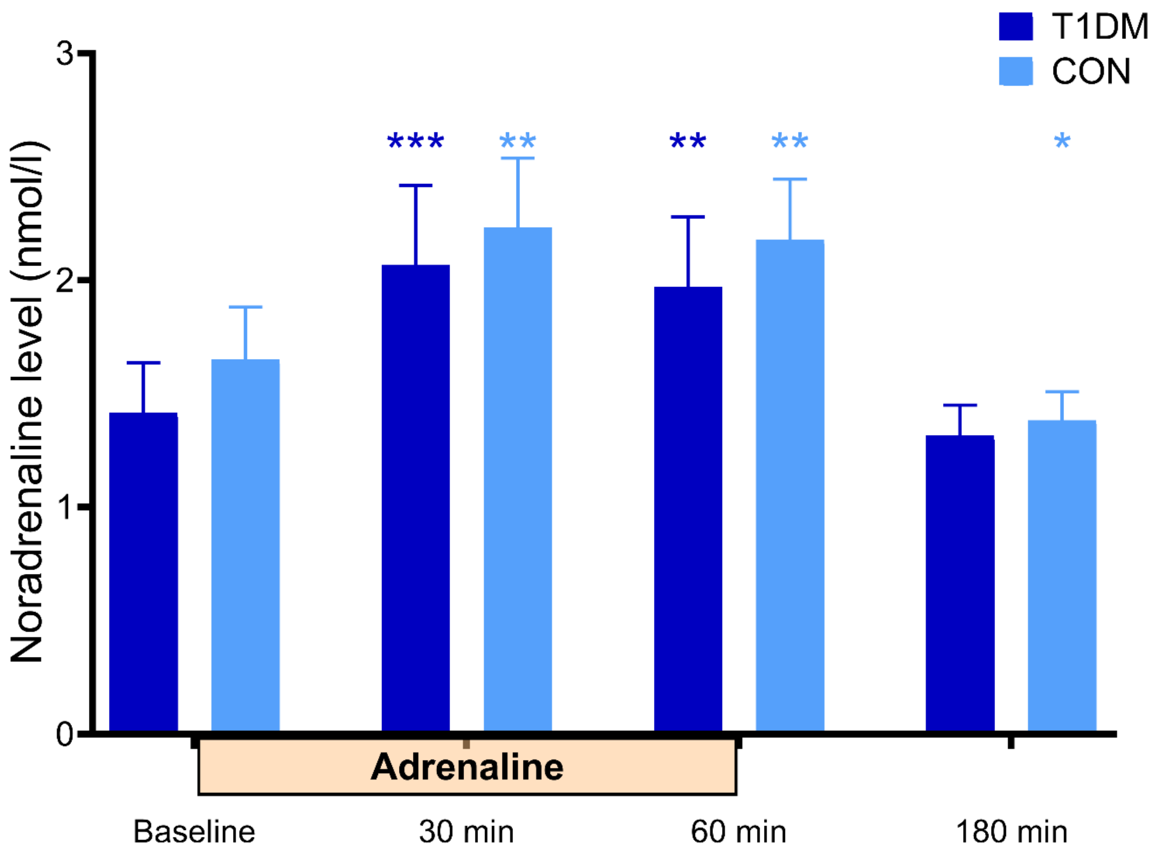

ESM Figure 2: *Noradrenaline levels in nmol/L after adrenaline administration for participants with type 1 diabetes mellitus (T1DM; dark blue bar, n=15) and matched control participants without diabetes (CON; light blue bar, n=15). Data are presented as mean ± SEM. *p <0.05, **p <0.01 ***p <0.001 versus baseline based on mixed model analysis.*

ESM Fig. 3a-c: *Ex-vivo* cytokine production by monocytes after stimulation with P3C following adrenaline administration

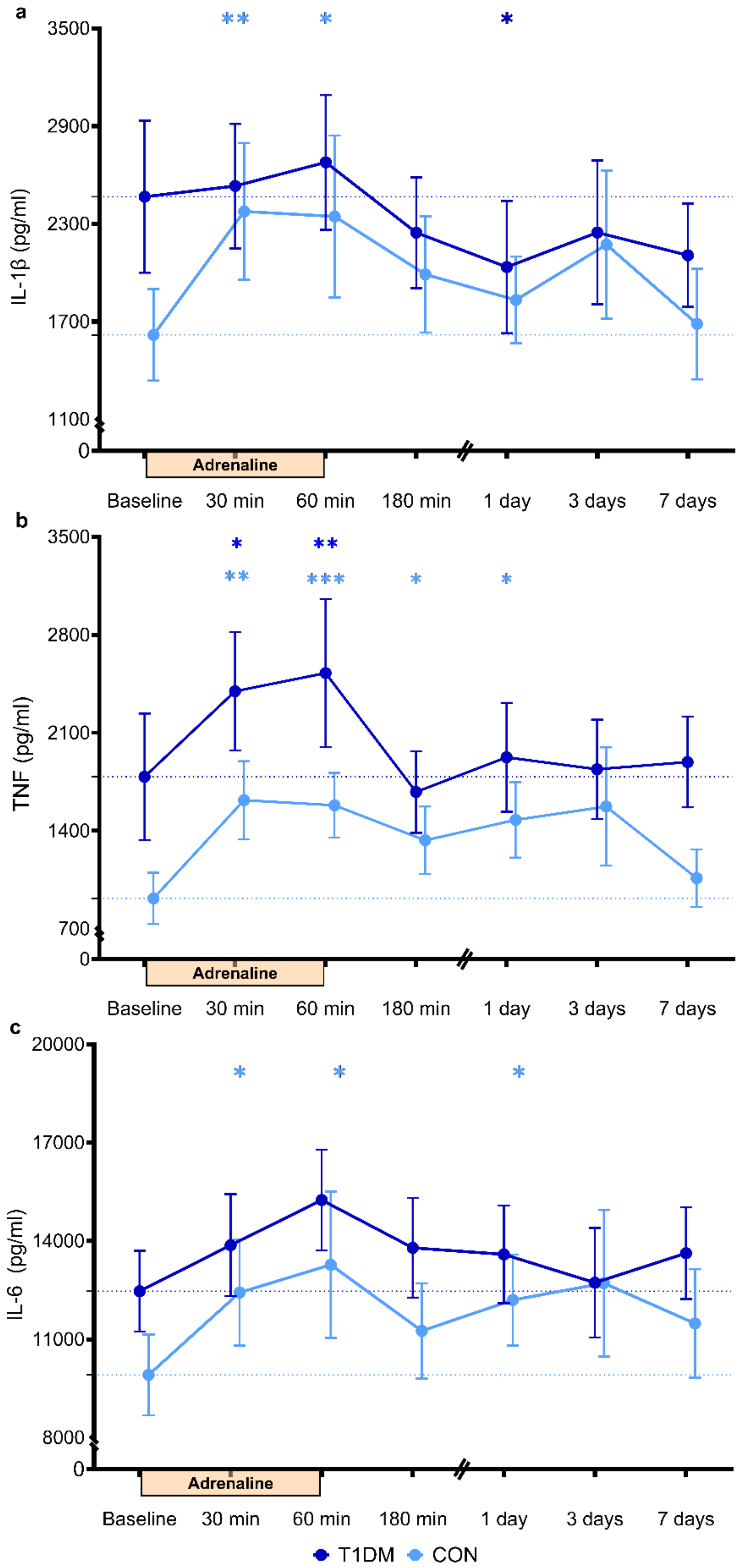

***ESM Figure 3**: Ex-vivo cytokine production by monocytes upon P3C stimulation after adrenaline administration. IL-1β (**a**), TNF (**b**), and IL-6 (**c**), for participants with type 1 diabetes mellitus (T1DM; dark blue, n=15) and matched control participants without diabetes (CON; light blue, n=15). Data are presented as mean ± SEM; *p <0.05, **p <0.01 and ***p <0.001 versus baseline based on mixed model analysis.*

ESM Fig. 4a-e: Proteomic analysis of 92 circulating inflammatory proteins

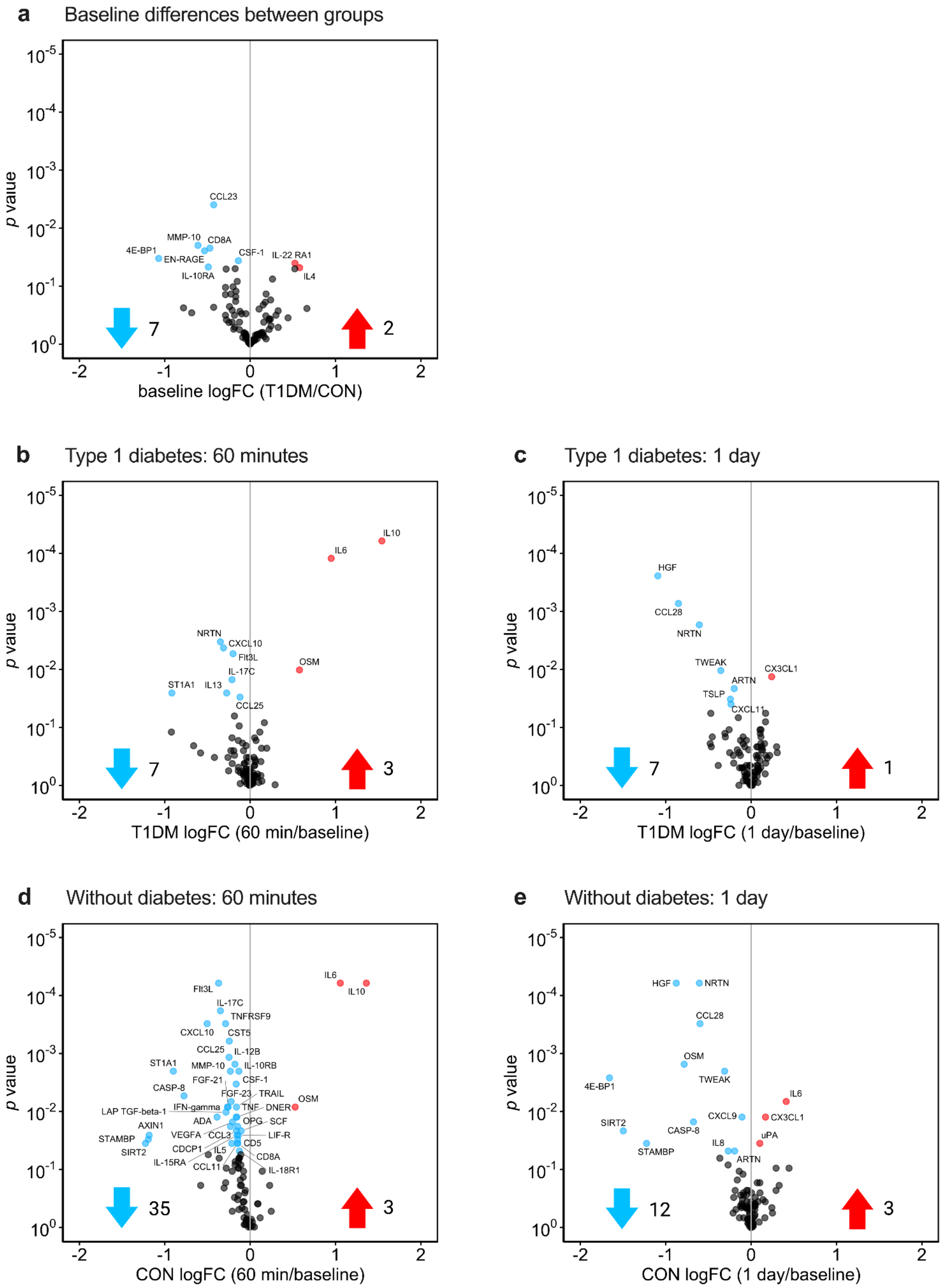

ESM Figure 4 a *Volcano plot of circulating inflammatory proteins comparing participants with type 1 diabetes mellitus [T1DM; n=15] and matched control participants without diabetes mellitus [CON; n=15[at baseline.* ***b-e****: Volcano plots of circulating inflammatory proteins per group and timepoint compared with baseline. Dots in blue represent significantly decreased proteins and dots in red represent significantly increased proteins (Wilcoxon paired test, unadjusted p-value < 0.05). The blue and red arrows represent the number of significantly decreased (blue) and increased (red) proteins. FC, fold change.*

ESM Fig. 5a-c: Circulating immune cell counts after adrenaline administration versus hypoglycaemic clamp

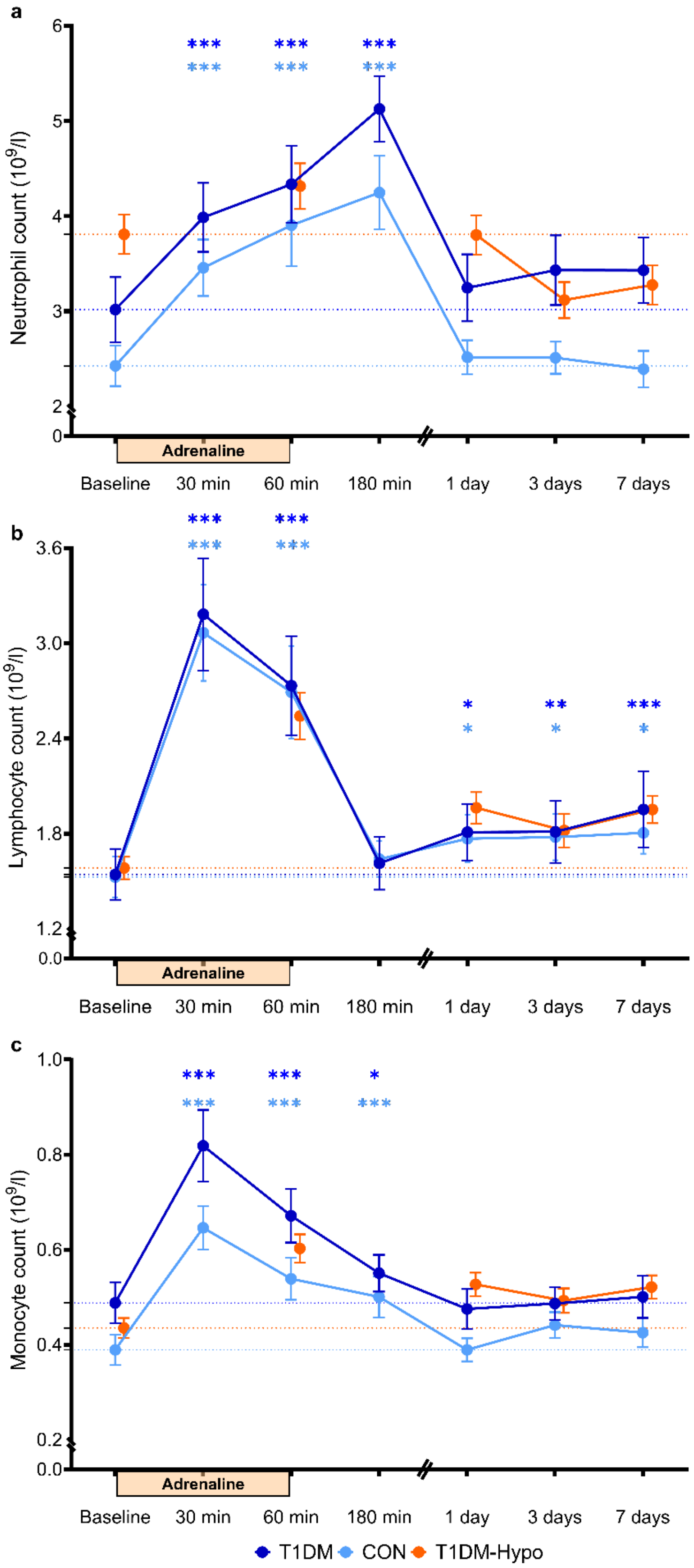

*__ESM Figure 5:__ Circulating immune cell counts after adrenaline administration versus hypoglycaemia. Neutrophil count (__a__), lymphocyte count (__b__), monocyte count (__c__), for participants with type 1 diabetes mellitus (T1DM; dark blue, n=15), matched control participants without diabetes (CON; light blue, n=15) and participants with type 1 diabetes mellitus after a hypoglycaemic clamp (T1DM-Hypo: Orange, n=47). The data visualized by the orange line (n=47) is data derived from a different cohort, (Verhulst and van Heck et al [8]) with recruitment and execution of experiments done in the period between August 2019 and March 2021. Data are presented as mean ± SEM; *p <0.05, **p <0.01 and ***p <0.001 versus baseline based on mixed model analysis. __a-c__: Timepoints 30 min and 180 min are missing for T1DM-Hypo as these measurements were not performed.*

ESM Fig. 6a-c: *Ex-vivo* cytokine production by monocytes after stimulation with LPS following adrenaline administration versus hypoglycaemia

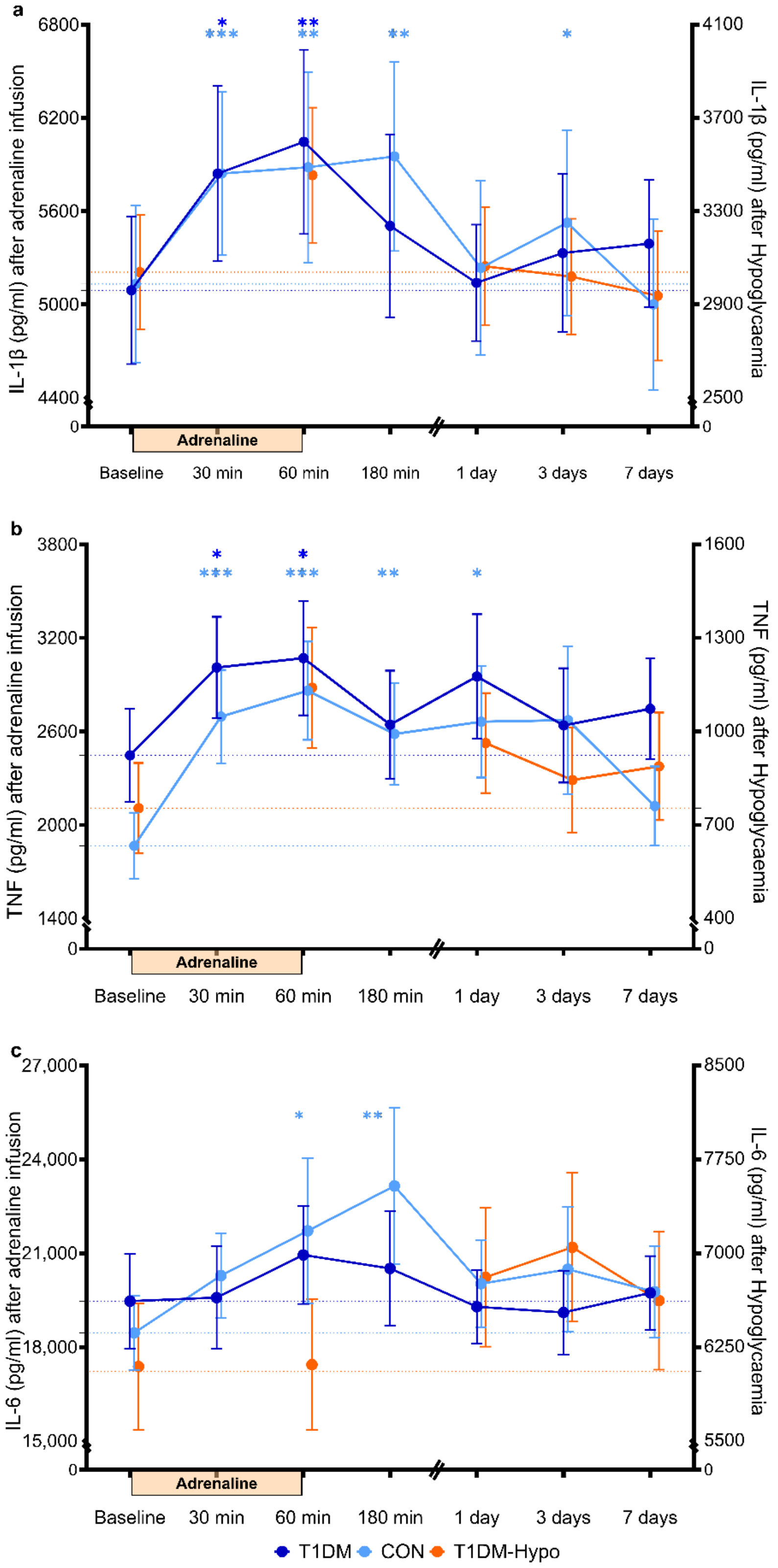

***ESM Figure 6**: Ex-vivo cytokine production by monocytes upon LPS stimulation after adrenaline administration versus hypoglycaemia. IL-1β (**a**), TNF (**b**), and IL-6 (**c**), for participants with type 1 diabetes mellitus (T1DM; dark blue, n=15), matched control participants without diabetes mellitus (CON; light blue, n=15) and participants with type 1 diabetes mellitus after a hypoglycaemic clamp (T1DM-Hypo; orange n=47). The data visualized by the orange line (n=47) is data derived from a different cohort, (Verhulst and van Heck et al [8]) with recruitment and execution of experiments done in the period between August 2019 and March 2021. Data are presented as mean ± SEM; *p <0.05, **p <0.01 versus baseline based on mixed model analysis. **a-c**: Timepoints 30 min and 180 min are missing for T1DM-Hypo as these measurements were not performed.*

ESM Fig. 7a-c: Ex-vivo cytokine production by monocytes after stimulation with P3C after adrenaline administration versus hypoglycaemia

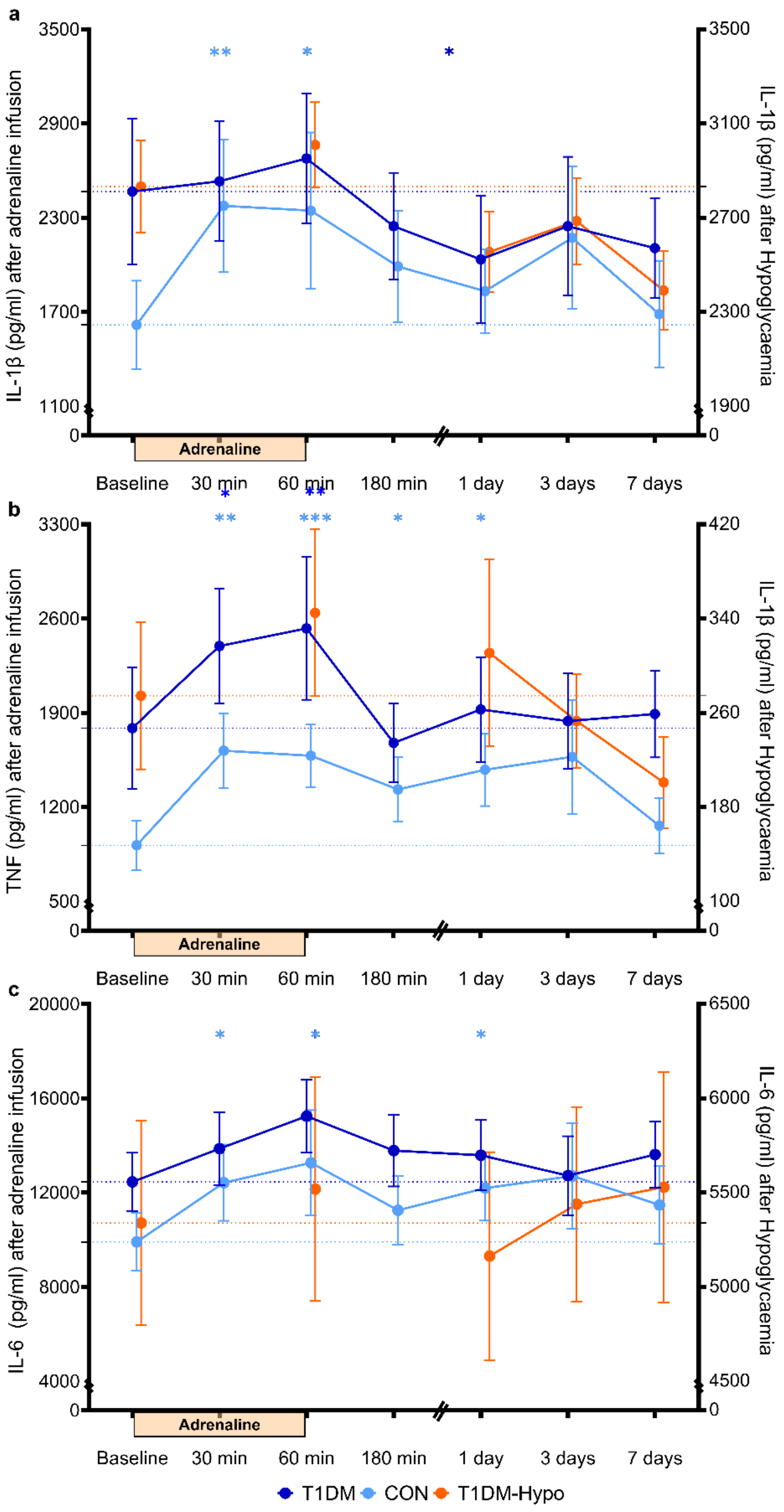

*__ESM Figure 7__: Ex-vivo cytokine production by monocytes upon P3C stimulation after adrenaline administration versus hypoglycaemia. IL-1β (__a__), TNF (__b__), and IL-6 (__c__), for participants with type 1 diabetes mellitus (T1DM; dark blue, n=15), matched control participants without diabetes (CON; light blue, n=15) and participants with type 1 diabetes mellitus after a hypoglycaemic clamp (T1DM-Hypo; orange n=47). The data visualized by the orange line (n=47) is data derived from a different cohort, (Verhulst and van Heck et al [8]) with recruitment and execution of experiments done in the period between August 2019 and March 2021. Data are presented as mean ± SEM; *p <0.05, **p <0.01 versus baseline based on mixed model analysis. a-c: Timepoints T=30 and T=180 are missing for T1DM-Hypo as these measurements were not performed.*

ESM Fig. 8: Hs-CRP levels after adrenaline administration versus hypoglycaemia

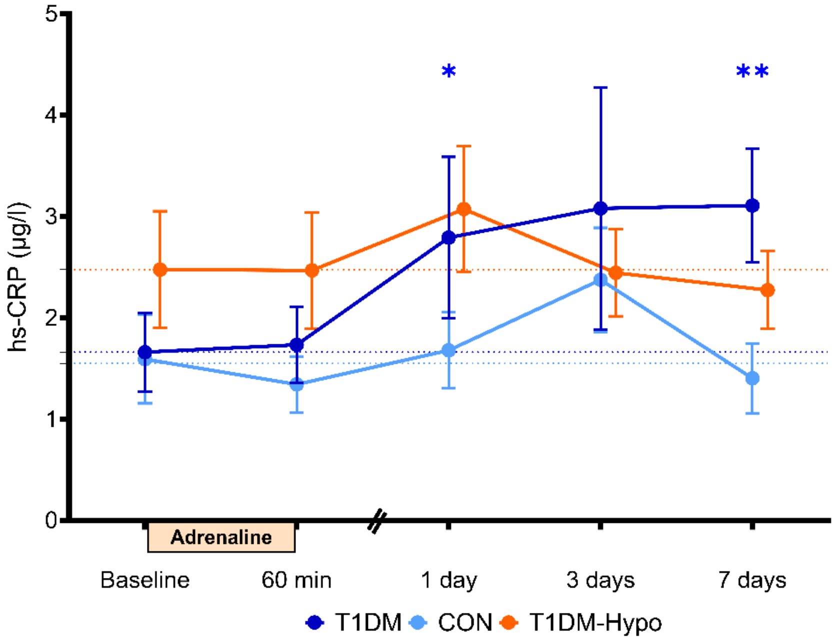

ESM Figure 8: *Circulating inflammatory protein hs-CRP after adrenaline administration versus hypoglycaemia for participants with type 1 diabetes mellitus (T1DM; dark blue, n=15), matched control participants without diabetes (CON; light blue, n=15) and participants with type 1 diabetes mellitus after a hypoglycaemic clamp (T1DM-Hypo; orange n=47) The data visualized by the orange line (n=47) is data derived from a different cohort, (Verhulst and van Heck et al [8]) with recruitment and execution of experiments done in the period between August 2019 and March 2021. Data are presented as mean ± SEM; *p <0.05, **p <0.01 versus baseline based on mixed model analysis.*

ESM Fig. 9: Circulating inflammatory proteins 7 days after adrenaline administration versus hypoglycaemia

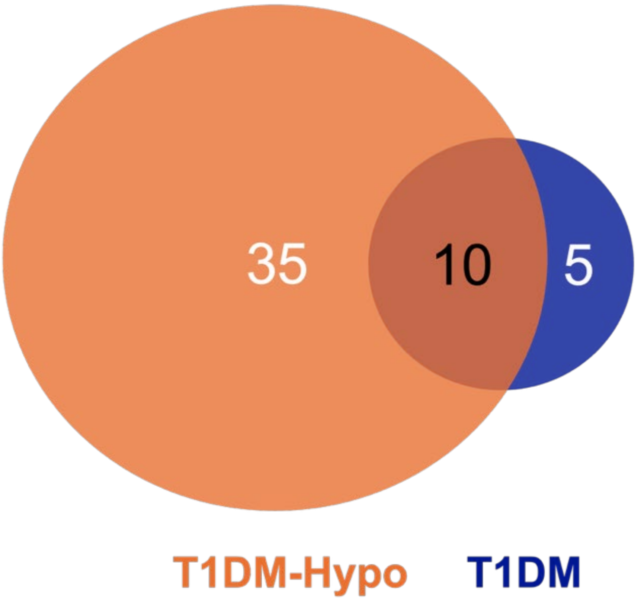

uPA	IL-18
Flt3L	CDCP1
VEGFA	CCL25
CX3CL1	CCL11
TNFRSF9	CST5

ESM Figure 9: *Circulating inflammatory proteins 7 days after adrenaline administration versus hypoglycaemia. Venn diagram showing significantly altered circulating inflammatory proteins (unadjusted p-value < 0.05) after 7 days in participants with type 1 diabetes mellitus after a hypoglycaemic clamp (T1DM-Hypo; orange n=47) and after adrenaline administration (T1DM; blue n=15). Overlapping proteins are listed in the accompanying table. The proteins in the T1DM-Hypo group (n=47) is data derived from a different cohort, (Verhulst and van Heck et al [8]) with recruitment and execution of experiments done in the period between August 2019 and March 2021.*

[1] Holding O Olink® Target 96 Inflammation Panels. Available from https://olink.com/products-services/target/inflammation/. Accessed 13-06-2023 2023
